# Supplementary material for: The development and structure of the mesentery
Source: Commun Biol. 2021 Aug 18;4:982. doi: 10.1038/s42003-021-02496-1 (PMC8373875; doi:10.1038/s42003-021-02496-1)
Supplement: Supplementary file 1 — Supplementary Information [file 42003_2021_2496_MOESM1_ESM.pdf]

Title:

The development and structure of the mesentery

Supplementary Information

Authors:

Kevin G. Byrnes [1, 2], Dara Walsh [1, 2], Leon G. Walsh [1, 2], Domhnall M. Coffey [1,2], Muhammad F. Ullah [1, 2], Rosa Mirapeix [3], Jill Hikspoors [4], Wouter Lamers [4], Yi Wu [5], Xiao-Qin Zhang [5], Shao-Xiang Zhang [5], Pieter Brama [6], Colum P. Dunne [2], Ian S. O'Brien [7], Colin B. Peirce [1], Martin J. Shelly [8], Tim G. Scanlon [8], Mary E. Luther[1], Hugh D. Brady [1], Peter Dockery [7], Kieran W. McDermott [2], J. Calvin Coffey [1, 2]

Affiliations:

[1] Department of Surgery, University of Limerick Hospitals Group, Limerick, Ireland.
[2] 4i Centre for Interventions in Infection, Inflammation and Immunology, School of Medicine, University of Limerick, Limerick, Ireland.
[3] Department of Anatomy and Embryology, Universitat Autònoma de Barcelona, Barcelona, Spain.
[4] Department of Anatomy & Embryology, Maastricht University, Maastricht, Netherlands.
[5] Digital Medicine Department, Biomedical Engineering College, Third Military Medical University, Chongqing, China.
[6] School of Veterinary Medicine, Veterinary Science Centre, Dublin, Ireland.
[7] Department of Anatomy, National University of Ireland Galway, Galway, Ireland.
[8] Department of Radiology, University of Limerick Hospitals Group, Limerick.

Corresponding author:

Name: Professor J. Calvin Coffey, PhD FRCS
Department of Surgery, University of Limerick Hospitals Group, Limerick, Ireland.
4i Centre for Interventions in Infection, Inflammation and Immunology, School of Medicine, University of Limerick, Limerick, Ireland.

Email: calvin.coffey@ul.ie

Contents

1. Supplemental Tables 4

2. Supplementary Note 1 (Model Validation) 6

3. Supplementary Note 2 (Embryology) 6

1.0. Development of the mesentery 6

2.0. Morphology of reconstructions of the mesentery at CS 13 6

3.0. The upper region of the mesentery during development 7

4.0. The mid region of the mesentery during development 7

5.0. The lower region of the mesentery during development 8

6.0. The developing mesentery and abdominal wall 8

7.0. “Displacement” and “coalescence” models of development 10

8 The intestine and mesentery during development 10

9.0. The pancreas and mesentery during development 11

10.0. Digestive system vasculature and mesentery during development 12

11.0. Narrative of key events during mesenteric development 12

4. Supplementary Note 3 (Anatomy) 13

1.0. Anatomy of the upper region of the *ex vivo* mesentery 14

2.0. Anatomy of the mid region of the *ex vivo* mesentery – the mid region switch 15

3.0. Anatomical basis of mesenteric continuity 15

4.0. Secondary folding along the right side of the mid-region fold 16

5.0. The mid region switch *in vivo* 16

6.0. Anatomy of the lower region of the *ex vivo* mesentery 17

7.0. The mesentery and abdominal wall 17

8.0. The upper region mesentery and abdominal wall 17

9.0. The mid-region mesentery and abdominal wall 18

10.0. The lower-region mesentery and abdominal wall 19

11.0. The position of the pancreas in the *ex vivo* mesentery 19

12.0. The position of the intestine in the *ex vivo* mesentery 20

13.0. The mesentery and abdominal digestive organ vasculature 21

14.0. Anatomical mechanisms connecting mesenteric and non-mesenteric domains 21

15.0. Comparative anatomy of the mesentery 22

16.0. Mechanisms of mesenteric development 23

16.1. Curve/buckle coupling during mesenteric development 23

16.2. Curve/buckle coupling in the *ex vivo* adult mesentery 23

16.3. Dynamic curve/buckle coupling in the *ex vivo* mesentery 23

17.0. Curve/buckle coupling and abdominal anatomy in the adult setting 24

17.1. Curve/buckle coupling and morphology of the mesojejunum and mesoileum.................. 24
17.2. *En masse* curve/buckling of the colon/mesocolon and taeniae coli.................................. 24
17.3. *En masse* curve/buckling and the mid-region switch 25
17.4. Curve/buckle coupling and the apex of the mid-region fold 25
17.5. Curve/buckle coupling and the under surface of the mid-region...................................... 26

1. Supplemental Tables

Software used	Function of software
Adobe Lightroom (version 8.1, Adobe®, San Jose, California, United States)	Image processing (histogram adjustment, including adjustments in brightness, contrast and sharpness)
ImageJ2 (TrakEM2 plugin, Fiji package distribution, v1.50e, U. S. National Institutes of Health, Bethesda, USA) [references]	Three-dimensional reconstruction from histological sections.
ZBrush (version 3.5 R3; Pixologic, California, USA)	Modelling and digital sculpturing of three-dimensional models generated by TrakEM2 plugin
Cinema4D (version 10.0, Maxon Computer GmbH, Germany)	Dynamic modelling and rendering of models

Supplemental table 1. Overview of software used.

Carnegie Stage	Sections	CRL (mm)	Sex	Fixation medium	Staining	Section plane	Section thickness (µm)
13	247	4.0	Indeter-minable	Mercury chloride + acetic acid	Alum cochineal (carmine)	Transverse	15
14	1109	6.7	Indeter-minable	Zenker's fixative	Haematoxylin and eosin	Transverse	10
15	558	9.0	Indeter-minable	Zenker's fixative	Haematoxylin and Eosin	Transverse	15
16	1103	10.5	Indeter-minable	Acetic Acid	Alum cochineal (carmine)	Transverse	8
17	1073	14.2	Indeter minable	Acetic Acid	Alum cochineal (carmine)	Transverse	10

18	837	14.0	Female	Acetic Acid	Alum cochineal (carmine)	Transverse	15
19	980	17.0	Male	Formalin	Azan and silver	Transverse	15
20	397	20.0	Male	Formalin	Alum cochineal (carmine)	Transverse	40
21	393	22.2	Female	Formalin	Alum cochineal (carmine)	Transverse	40
22	2090	28	Male	Formalin	Haematoxylin and Eosin	Transverse	10
23	2151	31	Male	Formalin	Azan	Transverse	12

Supplemental table 2. Overview of digitized datasets of embryos used in study. All embryos were sourced from the Carnegie collection, US.

**Weeks post fertilisation**	**Collection**	**Sex**	**Fixation medium**	**Staining**	**Section plane**
9 weeks	LUMC	Male	Formalin	Haematoxylin and Eosin	Transverse
9.5 week	LUMC	Male	Formalin	Haematoxylin and Eosin	Transverse
10 weeks	AMC	Male	Formalin	Haematoxylin and Eosin	Transverse
Full term	C-VHP	Male	Frozen	None	Transverse

Supplemental table 3. Overview of digitized datasets of foetuses used in study.

2. Supplementary Note 1 (Model Validation)

Artefact can arise in developing three dimensional models, and lead to misinterpretation of morphology. To assess the accuracy of reconstructions of the developing mesentery, we compared these with reconstructions of "mesenteric mesenchymal masses" by De Bakker *et al.*[1]. Reconstructions of both were similar, and at each time point examined. To further assess accuracy we compared reconstructions of the mesentery, with those of the developing digestive vasculature (Supplementary Fig. 1). The developing vasculature aligned with mesenteric morphology. The accuracy of reconstructions was further reflected morphological similarities between models of successive stages of mesenteric development.

As part of the study, we excised the intact mesentery, from adult human cadavers. Dissection may alter morphology (and hence interpretations of this). To assess if this had occurred, we compared the shape of the *ex vivo* and *in vivo* mesentery (Supplementary Fig.1). The morphology of each was similar at all craniocaudal levels. In addition, the regional anatomy of the *ex vivo* mesentery matched clinical observations during surgery, in radiological interpretations of the abdomen, and during post mortem using the Rokitanski approach[2]. Collectively the findings mean the shape of the *ex vivo* mesentery corresponds with that of the *in vivo* mesentery.

3. Supplementary Note 2 (Embryology)

Digital models of the developing mesentery and digestive organs have been compiled in Supplemental Atlas Section 1. Digital models relevant to each figure (main and supplemental figures) were compiled in Supplemental Atlas Section 2. All models are interactive.

1.0. Development of the mesentery

The morphology of the developing mesentery underwent little investigation prior to this study. This is likely due to the challenges involved in inferring 3D shape from 2D histological slides. 3D reconstructions of the developing mesentery overcome that challenge by enabling direct inspection of shape (Fig. 1). The following are descriptions of 3D reconstructions of the developing mesentery and associated organs.

A mid-region fold developed early with the result that the mesentery was subdivided into upper (pre-fold), mid (fold) and lower (post-fold) regions (see below)(Fig. 1c-f). Distinct anatomical boundaries were not apparent between regions. However, this format of regionalisation was apparent at all stages, including in the adult setting. As a result, it was possible to follow each region temporally, determining and comparing shape at and between successive time points.

2.0. Morphology of reconstructions of the mesentery at CS 13

At CS 13 the upper region of the mesentery spanned the coelom from anterior to posterior wall, in the midline sagittal plane (Fig. 1). The mid and lower regions lacked direct connection with the anterior abdominal wall and thus had a free anterior border. All regions were continuous with the abdominal wall in the posterior midline. The developing liver was directly connected to the upper region. Given its relative bulk, it obscured most of upper region from view. To

help visualise the mesentery, the liver was subtracted from digital models. When this was completed for CS 13, an indentation was apparent in the right side of the upper region (Fig. 1a).

3.0. The upper region of the mesentery during development

The findings demonstrated that the indentation in the right side of the upper region progressively invaginated deeper into the upper region. The upper region thus acquired a sac-like appearance, comprised of neck (at the indentation) and body sections. With further development, the upper region sac overlapped the upper and left lateral surfaces of mesentery distal to it (i.e. the mid-region)(Fig. 1). Although some of these properties had previously been noted, a detailed characterisation of the morphology of the neck is lacking[3,4]. From CS 15 onwards, the neck of the upper region comprised superior and inferior mesenteric arches (Fig. 1, Supplementary Fig. 1k-n). These merged anteriorly and posteriorly completing a channel to the cavity of the sac. The anterior confluence extended to the undersurface of the liver. The superior arch continued left laterally, as the anterior and posterior walls of the sac at that level. The inferior arch continued left laterally as the posterior, inferior and anterior walls of the sac at that level. The inferior arch also continued distally as the right side of the mid-region fold and providing the anatomical continuity between upper and mid-regions of mesentery.

The cavity of the upper region sac was slit-shaped (Fig. 1b). The boundaries of the cavity were marked by a recess formed at the junctions of the sac walls. A superior recess occurred at the junction between anterior and posterior walls. An inferior recess occurred at the junction between the inferior and anterior walls. The superior and inferior recess merged laterally. Similar anatomical properties were apparent in the adult setting (see below).

With continued development, the upper region sac expanded laterally and anteriorly, to overlap the mesentery distal to it (i.e. the mid-region fold) (Supplementary Fig. 2a-e). At week 10, the inferior wall of the sac was apposed to the upper surface of the mid-region fold. A plane of separation was apparent between apposed surfaces. This organisation was apparent in the full term fetal and adult mesentery.

4.0. The mid region of the mesentery during development

Although originally demonstrated Bardeen (over a century ago) the mid-region fold underwent little investigation since then[5]. The following is a description of the morphology of the mid region during development. At CS 15, the mid-region fold comprised central and peripheral zones (based on the proximity of these to the posterior abdominal wall) (Fig. 1c-f). The superior mesenteric artery divided the fold into right and left sides. The proximal pole (i.e. start point) of the fold corresponded to the junction between the neck of the upper region and right side of the central zone. The distal pole (i.e. end-point) of the fold corresponded to the junction between the left side of the central zone and lower region of the mesentery (Fig. 1c-f). Within the mid-region fold, the primary intestinal loop tracked from central to peripheral zones, first in the right side of the fold, turning at the apex, then returning centrally in the left side of the fold. The above description held for all successive stages examined.

At CS 18 the left side of the peripheral zone was cephalad its opposite right side (Fig. 1g-o). The sides of the central zone were in the original position, i.e. on either side of the SMA. Given endoderm differentiated from associated mesentery, the sides of the mid-region fold could be tracked by following the position of adjoining intestine (Fig. 1g-o). The position of the original right side of the mid-region fold could thus determined by following the duodenum, jejunum and ileum. The position of the original left side of the mid-region fold could be determined by following the right colon, and the hepatic and splenic components of the transverse colon. At week 10, the duodenum was on the right side of the SMA, which meant that the original right side of the central zone was also on the right side of the SMA. The jejunum and ileum were positioned on the left side of the SMA, which meant the mesojejunum and mesoileum were also on the left. At that stage, the right and hepatic region of the transverse colon and adjoining mesentery were on the right side of the artery. The splenic component of the transverse colon was centrally on the left side of the artery, as was the adjoining mesentery.

5.0. The lower region of the mesentery during development

Descriptions of the developing lower region of mesentery focus on lateralisation, fusion and regression of this region. In keeping with this, the left mesocolon and mesorectum are frequently described as "misnomers." Given descriptions of the morphology of the developing lower region are lacking, we characterised the shape of the lower region during development. To first expose the lower region, the upper region was digitally hinged upwards and off the left side of the central zone (Supplementary Fig. 2a-e). At CS 16, the lower region extended from the left side of the central zone distally, to the termination of the mesentery. The unattached anterior border of the mesentery encased developing endoderm. During development, the intestine gradually differentiated (or "emerged" from) from adjoining mesentery (Fig. 2,3). Garcia-Arraras determined the cellular and molecular basis of this process in lower order species[6,7]. Differentiation of the intestine meant that by week 10, components of the lower region could be named based on the adjoining intestine. Progressing cranio-caudally, the lower region comprised left mesocolon, mesosigmoid and mesorectum (Supplementary Fig. 2a-e).

At week 10, the inferior wall of the upper region overlapped the central zone of the mid-region (Supplementary Fig. 2a-e). Caudal to this overlap, the left mesocolon was apposed to the posterior abdominal wall (Fig. 2). The mesosigmoid was apposed medially but not laterally. This organisation meant the borders of the lower region changed from anterior and posterior positions, to lateral and medial positions respectively. Nearer the pelvis, the medial and lateral borders of the mesosigmoid converged towards the midline and continued distally into the pelvis as the mesorectum. The mesorectum encased developing rectum.

6.0. The developing mesentery and abdominal wall

As demonstrated above, multiple organs are directly connected to the mesentery during development (Fig, 3a). This means that the relationship between mesentery and abdominal wall (and how this develops) is relevant to the function of these organs. Conventional descriptions hold that the mesentery maintains a direct midline connection with the posterior abdominal wall. From that connection, it hinges laterally, in a process termed lateralisation.

There are several limitations to that description. It lacks an anatomical correlate, a hinge-like region of mesentery has yet to be identified. The description does not explain the relationship between the transverse mesocolon, (or small intestinal mesentery) and posterior abdominal wall. Given this, the anatomical junction between mesentery and abdominal wall (and how this develops) requires re-investigation. Knowledge of the morphology of the developing mesentery facilitates this.

The following focuses on the relationship between the lower region of mesentery and posterior abdominal wall. Similar observations applied at upper and mid-regions of the mesentery (Fig. 2). At CS 13 a mesothelial continuity was apparent between the surfaces of the mesentery and posterior abdominal wall. At this junction the mesothelium was reflected from one structure to the other. Beneath the mesothelium, mesodermal mesentery was continuous with the posterior abdominal wall. Vascular continuity was also apparent between developing vessels within the mesodermal mesentery and posterior abdominal wall (Fig. 3).

At CS 18, a demarcation was apparent between mesodermal mesentery and posterior abdominal wall (Fig. 2). This indicated that whilst these structures had separated, they remained apposed. Continuity of the surface mesothelium was still apparent. The left-sided mesothelial reflection marked the left-sided extremity of demarcation. The right mesothelial reflection marked the right-sided extremity. A similar organisation was apparent at all successive stages up to the full term fetal and adult stage (see below).

At CS 21, demarcation of mesodermal mesentery and posterior abdominal wall was again apparent and limited in distribution to the midline (Fig.2). Demarcation was evident from the oesophago-gastric junction to the insertion of the coeliac trunk into the mesentery (Fig. 2). It was apparent from the insertion of the superior mesenteric artery to that of the inferior mesenteric artery. Demarcation was observed between developing mesorectum and surrounding abdominal wall.

After CS 21, demarcation was apparent on either side of the midline (Fig. 2). At the level of the upper and lower regions of the mesentery, demarcation was evident to the left of the midline. At the mid-region level, it was apparent to the right of the midline. The right and left reflection marked the lateral limits of demarcation. Where demarcation was evident to the left of the midline, the left reflection was displaced to the left of the midline. Where demarcation was apparent to the right, the right reflection was displaced right.

At week 10, demarcation was apparent between the central (but not the peripheral) zone of the mid-region fold, and posterior wall. The right mesothelial reflection marked the right lateral limit of demarcation. A comparison of successive stages suggested that the zone of demarcation then progressively included the peripheral zone of the mid-region fold. The process progressed obliquely from the central region of the abdomen, to the right iliac fossa. In keeping with this, the right reflection was progressively displaced in the same direction, marking the limit of demarcation. Similar relationships were apparent in the adult setting (see below).

7.0. "Displacement" and "coalescence" models of development

The "coalescence" model of development holds that the mesentery and posterior abdominal wall remain directly connected in the posterior midline. From there, the mesentery hinges to the side (i.e. lateralises), then apposes (fuses) with the wall and then regresses (regression)[8,9]. In keeping with this, the right and left mesocolic and mesoduodenal areas of mesentery become vestigial, and organs that had adjoined these earlier during development, become "retroperitoneal," in position. The model implies that some organs change from "intra" to "retro" peritoneal in position. Coalescence was supported by Langer, Meckel, Toldt and Klebs[10,11]. Other anatomists (mainly Treitz, Luschka, Muller, and Waldeyer) argued in favour of a "displacement model," whereby the final organisation of the peritoneum was determined by its displacement. As a mechanical basis for displacement was not apparent, and the coalescence model was accepted.

The findings in this study provide a mechanistic basis for displacement (Fig. 2). They indicate that mesodermal mesentery first separates from the posterior abdominal wall (demarcation), but that these remain apposed. Next, mesentery nearer the posterior abdominal wall adheres (adhesion) to the latter. This process is directional, occurring from medial to lateral beneath the surface mesothelium. As a result, the surface mesothelium at the junction between mesentery and abdominal wall is displaced.

8 The intestine and mesentery during development

Given that multiple digestive organs develop on or in the mesentery, and the morphology of the mesentery was now apparent (see above), it was possible to describe the position of each organ in mesenteric terms. There are several benefits to this approach. It means that organ position can be described relative to a single frame of reference. If the position of multiple organs is determined based on the same frame of reference, then the positional relationship between these, can also be established. The following is a description of the mesenteric position of the intestine during development.

Both the stomach and rectum developed *within* mesentery (Fig. 3a, Supplementary Fig. 2f-i). At completion of development, these were encased posteriorly in adjoining mesentery (as if "carried *on"* mesentery). Between gastroduodenal and rectosigmoid levels, intestine developed *at* the periphery of the mesentery. The duodenum developed at the right side of central zone of the mid-region fold. The jejunum and ileum developed at the periphery of the original right side of the fold. The right and transverse colon developed at the periphery of the original left side of the fold. The left colon and sigmoid developed at the periphery of the lower region of the mesentery.

The findings mean that at completion of development, the gastroduodenal junction corresponds to a transition zone at which the anatomical relationship between intestine and mesentery changes. Proximally, intestine (i.e. stomach) is *on* the mesentery. Distally, the intestine (from duodenum onwards) is positioned *at* the periphery of the mesentery (Supplementary Fig. 2i). A further transition occurs at the rectosigmoid junction. The intestine (sigmoid) changes from being positioned *at* the periphery of the mesentery (as the sigmoid colon), to *on* the mesentery as the rectum. The upper two thirds of the rectum were encased posteriorly and posterolaterally

whilst the distal rectum was circumferentially surrounded in mesentery. Similar relationships were apparent in the adult setting (see below).

The anatomical relationship between mesentery and intestine influences the position of the intestine. At week 10, the right side of the mid region fold was centrally to the right and peripherally to the left of the superior mesenteric artery (SMA) (Fig. 1, Supplementary Atlas Sections 1,2). This meant the adjoining intestine was similarly positioned; the duodenum was on the right of the SMA, while jejunum and ileum were peripherally on the left. At week 10, the left side of the mesenteric fold was peripherally on the right and centrally to the left of the SMA. In keeping with this, the adjoining intestine was similarly positioned; the right colon and hepatic flexure were on the right side of the SMA, while the splenic flexure was positioned centrally on the left side.

9.0. The pancreas and mesentery during development

Clarification of mesenteric morphology meant it was possible to describe the position of the developing pancreas, in mesenteric terms. A comparison of the mesenteric position of the pancreas, intestine and supporting vasculature in turn resolved the anatomical relations of the pancreas. This is explained as follows.

At CS 13, ventral and dorsal buds of the pancreas were intra-mesenteric, distal to the neck of the upper region (Fig. 3). At that level, they were located anterior and posterior the developing endoderm. At CS 15, the ventral pancreatic bud was located in mesentery forming the right side of the central zone of the mid-region. The dorsal bud commenced in the junction between upper and mid-region. From there it continued left laterally in the posterior wall of the upper region sac.

From CS 18 onwards, the ventral pancreatic bud was encased posteriorly by mesentery forming the right side of the central zone. The nearby dorsal bud was encased posteriorly by mesentery at the junction between regions. From there the dorsal bud extended laterally on the posterior wall of the upper region sac.

At CS 21 and CS 23 stages, the upper region sac had expanded left of the midline and overlapped the upper and left surface of the mid-region fold (Fig. 3). As the superior mesenteric artery and vein were located in the ridge of the fold (see below) the upper region sac appeared to arch over these vessels. Given the mesenteric position of the pancreas, it also arched over the superior mesenteric artery and vein. Importantly, the neck of the pancreas was encased posteriorly in mesentery at the junction between upper and mid regions. The superior mesenteric vein continued proximally as the portal vein, in the junction. This organisation explained the anatomical relationship of the pancreas, portal and superior mesenteric veins. A similar anatomical relationship was apparent in the adult setting.

10.0. Digestive system vasculature and mesentery during development

Little is known regarding the mesenteric position of the vasculature of the developing digestive system. Given that we had clarified the shape of the mesentery during development, it was now possible to determine the position of the vasculature within the mesentery.

At CS 13, three arterial trunks were apparent at the anterior surface of the abdominal aorta. These inserted into the posterior aspect of the mesentery, without branching. *Intra* mesenteric branching was not apparent. At CS 15, three arterial trunks were apparent (Fig 1; Supplementary Fig. 1b-g). The middle trunk was located immediately distal the upper trunk. The distal trunk was positioned more caudad. All three inserted into the posterior aspect of the mesentery. Going from cephalad to caudad, these corresponded to the coeliac trunk (CT), superior and inferior mesenteric arteries (SMA and IMA respectively). Distal to its insertion into the mesentery, the SMA continued antero-inferiorly in the ridge formed by the junction of the right and left sides of the mid-region fold **(**Supplementary Fig. 1b-g**).** The superior mesenteric vein was to the right of the artery. The superior mesenteric vein continued proximally in mesentery posterior to the neck of the pancreas, At that level it was termed the portal vein.

The mesenteric position of digestive organ vasculature was similar at CS 15,16 and 17 **(**see Atlas**).** At CS 18, intra-mesenteric branching of the coeliac trunk was apparent, and aligned with the topography of the upper region sac. The splenic division extended laterally in the posterior wall to the spleen (Fig 1, Supplementary Fig. 1b-g). The common hepatic artery was located first in the floor of the inferior arch of the neck. From there it entered the inferior border of the anterior confluence of the neck, where it was adjacent the portal vein. Mesenteric and vascular anatomy were aligned at CS 20, 21 and 23 stages. At CS 23, an inferior mesenteric vein was apparent in the lower region of mesentery in which it continued proximally towards the left side of the mid-region fold.

11.0. Narrative of key events during mesenteric development

Continuity between developing mesentery and digestive organs means that the development of the shape of the mesentery influences how abdominal digestive organs are distributed within the abdomen. A narrative explaining key events in mesenteric development, is lacking. We used preceding observations to generate a narrative and videos depicting the development of the mesentery (Supplementary Atlas Sections 3-5).

The following narrative is a synopsis of key, organ-level events during mesenteric development. At the CS 13 starting point, the mesentery comprises upper, mid and lower regions, anterior and posterior borders, right and left sides. It includes a densely packed stroma (mesodermal mesentery) covered on either side by mesothelium. Each component of the mesentery is continuous with a corresponding element in the posterior abdominal wall.

Early during development, differential lengthening of intestine and adjoining mesentery results in the intestine forming a loop (primary intestinal loop). The mesentery forms a fold at the same level. At CS 15, the fold divides the mesentery into upper (pre-fold) region, mid (fold) and lower (post-fold) regions. After CS 17, the upper region sac expands laterally, to the left,

and antero-inferiorly. As a result, it overlaps the upper and left surface of the mid-region fold distal to it.

The right side of the mid-region fold undergoes extensive intestino-mesenteric curve/buckle distortion. In contrast, the entire left side (colon and mesocolon) distorts *en masse.* Curve/buckle formation of the intestine and mesentery on the left is guided radially and counter-clockwise around the origin of the middle colic artery. Torque and displacement forces pull the opposing right side of the mid-region fold to the left of the midline. With continued colonic lengthening and mesenteric buckling, the remainder of the mid-region fold progressively unfurls towards the apex of the fold. After this event, the original right side of the fold commences centrally on the right and continues peripherally on the left of the SMA. The original left side commences peripherally on the right and returns centrally to the left side of the SMA. This means that between the central and peripheral zones of the fold, the left and right sides change from aligned to non-aligned (i.e. they have switched). If this process fails the sides remain aligned. This is observed in malrotation, a congenital condition that classically is described in intestinal terms.

Between CS 15 and 17, the mesodermal mesentery and posterior abdominal wall separate but remain apposed. Mesothelial continuity is retained at and between the surfaces of both. Next, mesodermal mesentery adheres to the posterior abdominal wall, from medial to lateral, beneath the surface mesothelium. At upper and lower mesenteric regions, adhesion occurs progressively, from the midline to the left. At the mid-region, adhesion first involves the central zone then progresses to involve the peripheral zone. As a result, the small intestine appears to be stacked into position, from jejunal to ileal levels. The mesothelial junction between mesodermal mesentery and abdominal wall is thus displaced left or right in tandem with adhesion. When adhesion of the mid-region is complete, the ileocaecal junction and adjoining mesentery are in the right iliac fossa, adherent to the posterior abdominal wall, beneath the mesothelium. If this process fails to occur (either completely or partially), the ileocaecal region remains mobile and can twist later in life. This is observed in patients with an "ileocaecal volvulus."

At completion of adhesion of the upper, mid and lower region of the mesentery, the shape in the adult setting is apparent. Further changes in shape occur at a regional, but not overall level, and are part of normal growth.

4. Supplementary Note 3 (Anatomy)

Digital models relevant to each figure (main and supplementary figures) have been compiled in Supplemental Atlas section 2. All models are interactive and can be thus be used to further explore the morphology in focus. Supplementary Atlas section 6 is a collection of videos that illustrate the anatomical points described in the following.

1.0. Anatomy of the upper region of the *ex vivo* mesentery

Normally, several physical barriers impede examination of the *in vivo* mesentery. As these did not apply *ex vivo,* we were able to characterise the regional anatomy of the entire mesentery (Fig. 3). The upper region of the *ex vivo* mesentery was sac-shaped, with neck and body sections (Supplementary Figs. 3,4). The neck comprised superior and inferior arches. These merged anteriorly and posteriorly to complete a mesenteric channel. The oesophago-gastric junction was at the superior arch. The gastroduodenal junction curved around the outer-surface of the inferior mesenteric arch. The inferior mesenteric arch continued distally as the right side of the central zone of the mid-region fold (i.e. mesoduodenum)(see below). The floor of the inferior arch formed the anatomical junction between upper and mid regions (Supplementary Atlas Sections 6.18-6.21). These findings closely matched observations related to the morphology of the developing mesentery.

According to the peritoneal-based model of abdominal anatomy, digestive organs are centrally connected by a network of peritoneal derivatives. Our characterisation of the regional anatomy of the mesentery demonstrated that many of these "derivatives" are components of the mesentery. The components of the *ex vivo* mesentery are summarised in schematic format in Supplementary Fig. 3. If a peritoneal-based terminology was apparent in conventional literature, this was included in the schematic. Cross-referencing mesenteric and peritoneal-based approaches in this manner demonstrates that if peritoneal-based labels are to be retained, our understanding of what these labels refer to should change. For example, the "greater omentum" corresponded to a region of the anterior wall of the upper region sac. The "lesser sac" corresponds to the main cavity of the upper region. The lesser omentum, corresponded to the anterior confluence of the upper region neck **(**Supplementary Figs. 3,4**)**. The anterior confluence overlapped the posterior surface of the stomach (Supplementary Figs. 3,4). It did not insert directly at the lesser curvature of the stomach. The portal vein, common hepatic artery and common bile duct were located at the inferior margin of the anterior confluence (Supplementary Figs. 3,4). This is conventionally considered a peritoneal structure, i.e. the "hepatoduodenal ligament."

Characterisation of the regional anatomy of the *ex vivo* mesentery clarified the anatomical nature of several structures. For example, the boundaries of the neck of the upper region formed a channel that opened into the main cavity (Supplementary Figs. 1k-n, 4e-f). The Foramen of Winslow is conventionally considered the anatomical entry to the cavity of the upper region. The cavity itself was enclosed by the walls of the upper region (Supplementary Figs.4,5). The upper limit of the cavity was marked by a recess formed by the junction between the anterior and posterior walls. This extended laterally towards the hilum of the spleen. From there, it curved medially into the inferior recess. The latter was formed by the junction between the inferior and anterior walls. The inferior recess ended medially near the head of the pancreas.

Conventional anatomy fails to explain why the upper surface of the transverse mesocolon is not directly apparent from within the lesser sac. This is explained by the anatomical relationship between the upper and mid region regions. During development, the expanding upper region overlaps the upper and left side of the central zone of the mid-region fold (Supplementary Fig.

2). The organisation is also apparent in the adult setting. It explains why, on entering the main cavity of the upper region the recesses of this are apparent, whilst the transverse mesocolon is not. In the *ex vivo* (i.e. adult) mesentery it was possible to separate the inferior wall of the upper region from the mid-region fold (Supplementary Fig. 4m-n, Supplementary Atlas Sections 6.18-21). Separation of these exposes the upper and left surfaces of the central zone (i.e. the transverse mesocolon) (Supplementary Fig. 4).

2.0. Anatomy of the mid region of the *ex vivo* mesentery – the mid region switch

We characterised the regional anatomy of the mid-region by examining its shape in the *ex vivo* mesentery. As was apparent for the developing mesentery, the mid-region in the adult setting was fold-shaped (Supplementary Fig. 6a-d, Supplementary Atlas Sections 6.18). This supports suggestions that *in vivo*, the mid-region of the adult mesentery is also fold shaped. Components of the mid-region could be named according to the region of adjoining intestine. These included meso-duodenum, meso-jejunum, meso-ileum and mesocolon. In turn this nomenclature facilitated description of the morphology of the fold. The fold had central and peripheral zones. It commenced centrally at the mesoduodenum, on the right side of the superior mesenteric artery (SMA**)**. It fanned out peripherally, as the mesojejunum and mesoileum, on the left of the SMA. The fold then continued peripherally as right mesocolon and the hepatic flexural region of the transverse mesocolon, on the right of the SMA axis. The hepatic flexural region returned centrally on the left of the SMA, as the splenic flexure. The latter continued distally as the left mesocolon. These observations mean the sides of the mid-region fold switch position relative to the superior mesenteric artery, between central and peripheral zones.

3.0. Anatomical basis of mesenteric continuity

Although mesenteric continuity has long been suspected, its anatomical explanation has remained elusive. Observations related to mesenteric development and regional anatomy in the adult, indicate that continuity occurs at the central zone of the mid region. To investigate this, we examined the regional anatomy of the central zone. The central zone comprised of right and left sides (i.e. on either side of the superior mesenteric artery (SMA)). The mesoduodenum formed the right side of the central zone (Fig. 4, Supplementary Atlas Section 6.21**)**. The splenic flexure formed the left side. A mesenteric continuity occurred between the inferior arch of the upper region, and mesoduodenum, i.e. the right side of the central zone. It was located posterior to the neck of the pancreas (which it encased) and contained the portal vein, common hepatic artery and common bile duct. It formed the floor of the inferior arch of the upper region neck (Fig. 4, Supplementary Atlas Section 6.21**)**. A mesenteric continuity was also apparent between the splenic flexure and the left mesocolon (i.e. lower region of the mesentery). The observations mean the central zone of the fold provided a structural continuity between upper and mid-regions (on the right of the SMA) and between the mid and lower regions (on the left of the SMA).

4.0. Secondary folding along the right side of the mid-region fold

We and others have suggested that a further mesenteric fold occurs at the junction between mesoduodenum and mesojejunum[4,12]. Folding at that level could explain several properties of abdominal anatomy. To investigate this, we examined the central region of the *ex vivo* mesentery and identified an additional (left to right) folding at the junction between mesoduodenum and mesojejunum (Supplementary Fig. 6, Supplementary Atlas Section 6.18-21**)**. This fold was limited to the right side of the mid-region fold. As such, it represented a *secondary* folding of the right side of the *primary fold* of the mid region fold. At the secondary fold, the right side of the mid-region fold changed orientation. At the central zone level it was oriented from right to left. At the periphery, it was oriented from left to right. This fold meant the periphery of the mid-region doubled back over the central zone. As a result, the mesocolon at the hepatic flexure overlapped and adhered to the mesoduodenal component of the central zone.

Secondary folding is of anatomical importance. It explains why, *in vivo*, the periphery of the mid-region fold is diagonally oriented from the centre of the abdomen to the right iliac fossa. Ordinarily, the diagonal orientation of the mesentery (from duodenojejunal to ileocaecal junction) is erroneously attributed to insertion of the small intestinal mesentery, into the posterior abdominal wall, and along this line Reference texts frequently describe the proposed attachment as the "root" or "attachment" of the small intestinal mesentery[13–15]. Primary and secondary folding also explains the appearance of the mesentery at the central region of the abdomen. This region is often termed the "root" of the mesentery. At that level, the mid-region fold transitions from central to peripheral zones. The appearance here is explained by secondary (left to right) folding along the original right side of the mid-region (i.e. primary) mesenteric fold.

5.0. The mid region switch *in vivo*

The mid-region switch has several positional implications. Given this, it is important to determine if it is present *in vivo*. To test this, we hypothesised it should be possible to directly visualise the mid-region switch in reconstructions of the *in vivo* mesentery. Examination of the latter confirmed a switch at the junction between mesodoudenal and mesojejunal components of the mid-region fold (Supplementary Fig. 6e-g**)**. To further investigate if the switch applied *in vivo*, we compared the positional anatomy of the inferior pancreaticoduodenal and first jejunal vessels. In patients with a normal intestinal (and hence mesenteric) conformation, the inferior pancreaticoduodenal and first jejunal artery were consistently observed to the right and left of the midline respectively (Supplementary Fig. 6h-k**)**. This means that the mesoduodenum and proximal mesojejunum were to the right and left of the SMA axis respectively. Collectively the findings indicate that *in vivo*, the right side of the mid-region fold switches position from right to left of the SMA, at the junction between mesoduodenum and mesojejunum.

6.0. Anatomy of the lower region of the *ex vivo* mesentery

The lower region of mesentery commenced at the left side of the mid-region fold, and continued distally as left mesocolon, mesosigmoid and mesorectum (see Atlas section 6). Prior to dissection, the lower region was apposed to the posterior wall of the abdomen, at the left mesocolic, medial mesosigmoidal and mesorectal levels. Lower region mesenteric anatomy has recently been extensively characterised[16].

7.0. The mesentery and abdominal wall

Given that all abdominal digestive organs are directly connected to the mesentery, the mesentery is the *primary* mechanism of connection of these to the body. In keeping with this, the anatomical relationship between mesentery and posterior wall of the abdomen is relevant to the function of all abdominal digestive organs.

We determined the anatomical relationship between the mesentery and the posterior abdominal wall. The surfaces of the *ex vivo* mesentery and abdominal wall could be subdivided into apposed and non-apposed (i.e. free) surfaces. The later were peritonealised, the former surfaces were not. At non-peritonealised surfaces, the mesentery and abdominal wall were flattened against each other, separated only by a thin connective tissue fascia (Fig. 5, Supplementary Atlas Section 6.16-18). The apposed surface of the mesentery comprised right and left sides (Fig. 5). The left side included the posterior surface of the upper region, splenic flexure, left mesocolon, mesosigmoid and mesorectum in that order (from top to bottom). The right side included the bare area of the liver, posterior surface of the mesoduodenum, and right mesocolon (from top to bottom), in that order.

A connective tissue layer (fascia) was apparent wherever a surface of the mesentery (and conjugate organs) was apposed to the abdominal wall (Fig. 5, Supplementary Atlas Section 6.16-17). A peritoneal-like reflection was evident at the periphery of the zone of apposition**.** The reflection marked the peripheral limit of apposition and the distribution of the fascia.

8.0. The upper region mesentery and abdominal wall

There is a lack of clarity regarding the relationship of the upper region of the mesentery and the posterior abdominal wall. Recognition of the sac-like regional anatomy of the upper region means it is now possible to clarify the anatomical relations between it and the surrounding abdominal wall in the adult setting.

Prior to dissection, the upper region of the mesentery was apposed to the abdominal wall (Fig. 5, Supplementary Atlas Section 6.16-6.17). A peritoneal-like reflection was apparent at the lateral limit of apposition of the upper region and abdominal wall. This region of reflection (arbitrarily termed the "lateral peritoneal reflection") bridged the lining of the free (i.e. un-apposed) surface of the upper region with that of abdominal wall. It commenced at the oesophago-gastric junction, from which it continued inferolaterally around the spleen. At the inferior pole of the spleen, it curved medially. It ended medially where the upper region sac and mid-region fold adhered. From there it returned laterally to the left of the midline, linking

the surface of the central zone (i.e. splenic flexure) and abdominal wall at the same level. It continued around the colic component of the splenic flexure, bridging the surface of this, and that of the posterior abdominal wall. It then extended distally along the lateral aspect of the colon where it marked the lateral limit of apposition of the descending colon and abdominal wall.

A further peritoneal-like reflection (the "medial reflection") bridged the lining of the free surface of the upper region neck, and that of the adjacent posterior abdominal wall (Fig. 5, Supplementary Atlas Section 6.16-6.17). This medial reflection extended from the oesophago-gastric junction to the insertion of the coeliac trunk into the mesentery. The topography of the regions of the reflection corresponded to that of the developmental mesothelial reflection, at the same level.

The reflection is of considerable surgical and pathological importance. Its division enables the surgeon enter and disrupt the plane formed by apposition of mesentery and posterior abdominal wall (see Atlas section 6). The reflection provides an important barrier against disease spread. The surface anatomy of the reflection determines the distribution of peritoneal fluid and any other fluids that may be present in the abdomen. Clarification of the macroscopic anatomy of the reflection thus has immediate implications for the clinician.

9.0. The mid-region mesentery and abdominal wall

Conventional descriptions maintain that the small intestinal mesentery inserts or attaches directly into the posterior abdominal wall, along a diagonal path from the duodenojejunal flexure to ileocaecal junction. Recognition of the shape of mesentery adjoining small intestine (i.e. the mid-region fold) prompts a revision of conventional descriptions. The following is a description of the anatomical relationship between the mid-region and posterior abdominal wall.

Prior to dissection, the periphery of the mid-region mesenteric fold was apposed to the posterior abdominal wall (Fig. 5, Supplementary Atlas Section 6.16-6.17). A connective tissue layer was interposed between mesentery (including conjugate organs) and the posterior abdominal wall. A peritoneal-like reflection bridged the surface lining of the fold and that of the adjacent posterior abdominal wall. It marked the limit of apposition of mesentery and abdominal wall, and the distribution of the fascia.

The reflection extended from the duodenojejunal flexure, diagonally across the posterior abdominal wall, to the right iliac fossa. It continued around the ileocecal junction, bridging the surface of the mid-region fold and abdominal wall. It extended proximally from the ileocaecal to the hepatic level, at the lateral aspect of the ascending colon. From there it curved medially. It ended centrally at the level at which the periphery of the mid-region overlapped the right side of the central zone. The peritoneal reflection marked the lateral limit of apposition of the mid-region mesentery to the posterior abdominal wall. The topographical distribution of the reflection correlated with that of the mesothelial reflection seen during development.

10.0. The lower-region mesentery and abdominal wall

Conventional descriptions hold that the left mesocolon is absent in the majority of individuals and that the mesosigmoid inserts directly into the posterior abdominal wall. In addition, adherence to the idea that the mesentery is a duplicature of peritoneum meant the left mesocolon and mesorectum were misnomers. These concepts persist in current reference texts. Clarification of the anatomy of the lower region of the mesentery (from left mesocolon to mesorectum) refutes these tenets and now enables a detailed characterisation of the anatomical relationship between the lower region and posterior abdominal wall, as follows.

A peritoneal-like reflection bridged the surface of the lower region mesentery (and conjugate intestine) and that of the posterior abdominal wall at the same level (Fig. 5, Supplementary Atlas Sections 6.16-6.17). The reflection tracked medially and diagonally from the junction between descending and sigmoid colon. It bridged the surface lining of the mesosigmoid and that of the posterior abdominal wall in the left iliac fossa. At the rectosigmoid junction it curved sharply caudad, to continue into the pelvis, where it bridged the surface lining of the mesorectum and that of the lateral pelvic side wall. It successively marked the lateral limit of apposition of the mesosigmoid and mesorectum, with the posterior abdominal wall.

A peritoneal-like reflection was also apparent at the medial boundary of the lower region mesentery, where it bridged the surface lining of the mesentery and that of the posterior abdominal wall (Fig. 5, Supplementary Atlas Sections 6.16-6.17). It extended from the duodenojejunal flexure distally, at the medial border of the left mesocolon and mesosigmoid, in that order. At the duodenojejunal flexure, it raised a fold of peritoneum. At the sacral promontory it continued into the pelvis bridging the surface lining of the mesorectum and that of the pelvic side wall.

Division of either the right or left lateral reflection exposed the plane formed by apposition of the lower region mesentery and posterior abdominal wall (Fig. 5, Supplementary Atlas Sections 6.16-6.17). This contained a connective tissue fascia. The topographical distribution of the fascia and reflection correlated with that of the zones of demarcation and mesothelial reflection seen during development.

11.0. The position of the pancreas in the *ex vivo* mesentery

The position of abdominal digestive organs is normally described in broad terms (intra, extra or retroperitoneal or based on the four quadrants) and is normally contextualised with reference to multiple, surrounding structures. This approach fails when the anatomy of related structures is variable, due either to congenital, surgical or pathological alterations. Given that all abdominal digestive organs develop on or in the mesentery, and remain connected to it, it is possible to describe the position of these with reference to a single reference frame, the mesentery (the mesenteric based approach). By cross-referencing mesenteric position, the relations of multiple organs and structures can be developed. In the following we demonstrate how the mesenteric-based descriptions of organ position, resolves several longstanding questions in abdominal anatomy.

The head of the pancreas was posteriorly encased (i.e."carried on") in mesoduodenal mesentery at the right side of the central zone of the mid-region fold (Supplementary Fig. 7). The neck was posteriorly encased by mesentery at the junction between the upper and mid-regions of the mesentery. The body was encased posteriorly in mesentery of the posterior wall of the sac.

The mesenteric position of the pancreas thus explains several morphological properties of the pancreas (Supplementary Fig. 7). The superior mesenteric artery and vein are positioned in mesentery over which the upper region (and pancreas) arch. As a result, when the pancreas is tracked anteroposteriorly in the coronal plane, it appears to arch over these structures. The portal vein is located in the same mesentery that encases the neck of the pancreas posteriorly. As a result, when the pancreas is followed supero-inferiorly in the axial plane, it spirals antero-inferiorly around the portal vein.

12.0. The position of the intestine in the *ex vivo* mesentery

The position of the intestine is normally described in general terms related to domains of the peritoneum and abdominal quadrants. These descriptions are further contextualized by referencing anatomically separate structures. For example, the position of the transverse colon is frequently described relative to the liver, duodenum, ileum, jejunum, stomach and spleen. As mentioned above, this approach is challenging and becomes even more so in the setting of physiological, congenital, surgical or pathological alterations. The relationship of the intestine and mesentery mean that it is now possible to describe the position of the intestine with reference to a single organ, the mesentery.

The double-spiral trajectory taken by the intestine is explained by its position at the periphery of the mesentery, which itself approximates to a double-spiral trajectory. The oesophago-gastric junction was posteriorly encased in mesentery at the superior arch of the upper region neck (Supplementary Figs. 4, 5). The gastroduodenal junction curved around the outer surface of the inferior arch. Between junctions, the posterior surface of the stomach was incompletely encased in mesentery. At the gastroduodenal junction, the intestine changed position from being *on* mesentery (i.e. at the stomach) to being *at* the periphery of the mesentery (i.e. duodenum) (Supplementary Fig. 7). The intestine between gastroduodenal and rectosigmoid junction was *at* the periphery of the mesentery. Distal to the rectosigmoid junction the rectum was posteriorly encased in mesorectum. The distal rectum is circumferentially encased in mesorectum. A similar morphology was apparent on examination of reconstructions of the developing mesentery (see above).

At the junction between duodenum and jejunum, the intestine followed the mesentery and folded sharply left to right (Supplementary Fig. 6). The mesojejunum and mesoileum were on the left of the SMA axis. This meant that the adjoining jejunum and ileum were similarly positioned. The right mesocolon was on the right of the superior mesenteric arterial axis, which meant the corresponding region of adjoining intestine (i.e. the right or ascending colon) was similarly positioned.

The hepatic component of the transverse mesocolon overlapped the right side of the central zone of the mid-region fold (Supplementary Fig. 7e-11, Supplementary Atlas Sections 6.18-

21). As the colon was adjoining periphery of the mesentery, the colic component of the hepatic flexure thus overlapped the right side of the central zone. This meant that it overlapped the mesoduodenum, duodenum and head of pancreas. The remainder of the transverse mesocolon (i.e. the splenic flexure) corresponded to the left side of the central zone and continued distally as the left mesocolon. In keeping with this, the adjoining transverse colon curved around the splenic flexure, as the colic component of the flexure, to continue distally as the left (i.e. descending) colon.

13.0. The mesentery and abdominal digestive organ vasculature

Normally, abdominal digestive organs are visually depicted as separated by an anatomical space. The vasculature associated with these organs occupies the spaces between them. The preceding observations demonstrate that space is occupied by the mesentery, and that the vascular circuitry of abdominal digestive organs is intra-mesenteric in position. Given this, and given clarification of mesenteric morphology, it is now possible to determine the mesenteric position of the vasculature of the abdominal digestive system. We characterised the mesenteric position of digestive system vasculature.

The coeliac, superior and inferior mesenteric arteries inserted into the posterior surface of the *ex vivo* mesentery, just distal to the aortic origin of each (Supplementary Fig. 8a-c). They did not branch prior to integrating in the mesentery. They subdivided within the mesentery. All branches were topographically aligned with surrounding mesentery.

The inferior mesenteric vein was located at the medial boundary of the left mesocolon. From there it continued into the splenic flexure (i.e. left side of the central zone of the mesenteric fold), in which it continued proximally to the apex of mid-region fold. At that level, the inferior mesenteric and splenic vein merged, continued anterior to the superior mesenteric artery to join the superior mesenteric vein. The resultant portal vein formed in the mesentery at the junction between the upper and mid-regions of the mesentery (see above) (Supplementary Fig. 8d-f).

Understanding the mesenteric relations of arteries of the abdominal digestive system enables localisation, and hence clinical treatment, of arterial diseases of the abdomen. It is also relevant to distribution of abdominal lymph nodes and vessels. Although not formally examined, the distribution of mesenteric lymph nodes and vessels follows that of arteries. In keeping with this, the mesenteric relations of digestive organ arteries also holds for digestive organ lymphatics. This has implications for our understanding of the dissemination of diseases that spread via lymphatic routes (i.e. solid organ malignancy). Mesenteric relations of the portal venous system have major implications for the localisation and treatment of venous diseases of the abdomen.

14.0. Anatomical mechanisms connecting mesenteric and non-mesenteric domains

Clarification of the anatomical foundation of the abdomen meant it was possible to characterise how the order at that level was maintained. To do this, we determined anatomical mechanisms by which domains are directly connected. The connections corresponded to the physical links that were disrupted during excision of the mesenteric domain (Supplementary Fig. 3,

Supplementary Atlas Section 6 (all)**)**. Central connections occurred at the origin at the major arterial trunks and at the junction between hepatic veins and inferior vena cava. These findings indicated that arterial inflow to the mesenteric domain occurs at the coeliac trunk, superior and inferior mesenteric arteries. They indicate that venous drainage occurs at the junction between the hepatic veins in inferior vena cava. A supplementary arterial inflow was sometimes apparent at the middle rectal artery level. Similarly, an alternative venous drainage was sometimes apparent at the umbilical vein (in the falciform component of the upper region).

Fascia provided an intermediate connection between domains. Fascia between apposed surfaces of both domains was not a *direct* connection as it was possible to separate fascia from overlying mesentery without disrupting either (Fig. 5, Supplementary Atlas Section 6.17). However, a direct connection was apparent at the origin of the major vascular trunks. Fascia coalesced around these to form a connective tissue collar conjoined with adventitia. This meant that at the aortic pole, fascia was continuous with aortic adventitia. At the mesenteric pole, fascia and vessels were topographically coupled at the insertion of these into the mesentery. Within the mesentery the fascia was conjoined with adventitia on the vascular side, and mesenteric interstitium on the opposing mesenteric side.

Peripherally the domains were directly linked by the peritoneal-like reflection (Fig.5, Supplementary Atlas Sections 6.16-17). This bridged the lining of the free surfaces of the mesentery, liver, spleen and colon on the *mesenteric* side, with that of the abdominal wall on the non-mesenteric side.

15.0. Comparative anatomy of the mesentery

Clarification of the development and shape of the human mesentery prompts reappraisal of these in the comparative setting. If similar findings emerged, this could have implications for our understanding of the organisation of the abdomen in animals in general.

Several properties were common to the animal species examined (Supplementary Atlas Section 7). Abdominal digestive system vasculature was mainly *intra* mesenteric in position, and regionally aligned with mesenteric anatomy. Lymph nodes were distributed along vessels within the mesentery. Adipose tissue surrounded mesenteric vessels with which it appeared conjoined. Perivascular fat was particularly prominent in the chimpanzee and human setting.

In general, the conformation of the mesentery differed between species. Given continuity between mesentery and abdominal digestive organs, this meant there were marked differences in the distribution of digestive system organs, between species. However, the conformation of the chimpanzee and human mesentery was similar (Supplementary Atlas Section 7). In keeping with this, the positional anatomy of digestive organs was similar in the chimpanzee and human setting.

As occurred in the human mesentery, a mid-region fold subdivided the mesentery in the chimpanzee into upper, mid and lower regions. In both settings, the mid-region mesenteric fold switched sides (relative to the superior mesenteric artery) from central to peripheral zones. In addition, the mesentery underwent a second (left to right) folding at the junction between mesoduodenum and mesojejunum with the result that the ileocaecal pole of the mid-region fold was positioned in the right iliac fossa. The anatomical mechanisms linking mesenteric and

non-mesenteric domains were similar in the chimpanzee and human. In both, detachment and disconnection of domains required division of the peritoneal reflection, separation of mesentery from underlying fascia, and division of the major vascular trunks (Supplementary Atlas Section 7).

16.0. Mechanisms of mesenteric development

Understanding shape meant we could investigate the mechanisms by which it arose. Differential lengthening of the intestine relative to adjoining mesentery generates mechanical forces that are determinants of gut development[17]. These forces arise when the mesentery and intestine are directly connected, and they manifest as curve/buckle distortion of the intestine and mesentery respectively (Supplementary Fig. 9)[18–20]. Given the above findings confirmed mesenteric continuity and intestinal connectivity, we investigated if the same mechanical forces could be important in mesenteric development, by examining for curve/buckle coupling.

16.1. Curve/buckle coupling during mesenteric development

We identified several instances of endodermal curve formation and in tandem mesenteric buckling (i.e. curve/buckle coupling) during development (Supplementary Fig. 9). For example, the mid-region fold corresponded to a single curve/buckle complex. Serial curve/buckling occurred on the right side of the fold. This explains the highly contoured appearance of the small intestinal mesentery in the adult. In contrast, an *en masse* mesocolic curve/buckling was apparent on the left side of the fold.

16.2. Curve/buckle coupling in the *ex vivo* adult mesentery

Curve/buckle coupling was apparent from gastroduodenal to recto-sigmoid junctions but not outside these limits. This is explained by the fact that between these junctions the intestine is located *at* the periphery of the mesentery. On either side, the intestine is posteriorly encased *by* adjoining mesentery (see above).

Extensive curve/buckle coupling was apparent at meso-jejunal and meso-ileal levels. In contrast, a single curve/buckle complex was apparent involving the right, transverse and left mesocolon. This is explained by *en masse* curve/buckling of the left side of the original mid-region fold.

Curve/buckle coupling was apparent in all animal species examined. An overlap occurred in the pattern of curve/buckle coupling in human and chimpanzee settings.

16.3. Dynamic curve/buckle coupling in the *ex vivo* mesentery

The *ex vivo* mesentery was a platform which enabled direct assessment of the effects of intestinal lengthening on mesenteric shape. Simple lengthening of the *ex vivo* small intestine between two fixed points led to curve formation of the intestine and buckling of adjoining mesentery. The curve of the intestine approximated to an Omega shape. Of note, continued

lengthening of an already curved intestine, led to progression from a curve/buckle to a coil/spiral complex. In the coil/spiral complex, the intestine was now alpha-shaped, while the sides of the mesentery formed a spiral. Similar shapes are observed *in vivo* in the pathological setting of volvulus (i.e. twisting of the intestine and mesentery around a narrow pedicle). The shapes are replicated during alpha-loop formation in almost every colonoscopy. Alpha-loop formation represents progression from a pre-existing curve/buckle complex to a coil/spiral complex. It occurs at sites of pre-existing curvature (i.e. at sigmoid, transverse and hepatic levels) when even limited advancement of the colonoscope leads to coil/spiral distorsion. If uncorrected for, it leads to pain and subsequent abandonment of the procedure. Understanding the anatomical basis of alpha-loop formation now enables the endoscopist take corrective actions, avoid causing pain and obviate abandonment of a procedure that requires considerable preparation on the patient's part.

Clock-wise rotation of the *ex vivo* colon returned the ileum and jejunum to the right of the superior mesenteric artery, and the colon and mesocolon to the left. Following this, extensive curve/buckling persisted at ileal and jejunal levels, but was absent at colonic and mesocolic levels. Counter-clockwise rotation of the colon then restored the normal colonic conformation and the mesocolic buckle.

The frequency with which curve/buckling was apparent at all stages of human life, and in many animal species, indicates that the forces generated by differential intestinal lengthening (on an adjoining mesentery) are as important a determinant of mesenteric growth and form as they are that of gut form.

17.0. Curve/buckle coupling and abdominal anatomy in the adult setting

Curve/buckle coupling explained several well established but hitherto incompletely explained properties of abdominal anatomy.

17.1. Curve/buckle coupling and morphology of the mesojejunum and mesoileum

In the *ex vivo* mesentery, the periphery of the original right side of the mid region fold comprises jejunum, ileum and adjoining mesentery. The intestinal margin of the mesentery is heavily contoured due to extensive curve/buckle coupling (see above). In contrast, the central region of mesentery is smooth. The transition from contoured to smoothened is explained as follows. The apex of each mesenteric buckle positionally corresponds to the origin of the end artery that supplies that buckle. Given each end artery originates close to the intestinal margin, the apex of each buckle is similarly positioned. As a result, the periphery of the mesentery is buckled, whilst the central region is smoothened.

17.2. *En masse* curve/buckling of the colon/mesocolon and taeniae coli

En masse curve/buckling of the colon/mesocolon (and not at the small intestinal level) is partially explained by the occurrence of taeniae coli in the colon. These are absent in the small intestine. They span the colon from the base of the appendix base to recto-sigmoid junction. They exert a break-like effect on colonic and mesocolic lengthening (when they are excised

the colon lengthens). It is feasible that the constraints imposed by the taeniae mean that when the colon does curve, the full length of the colon is incorporated in that curve, with the result that the entirety of the mesocolon is similarly involved.

17.3. *En masse* curve/buckling and the mid-region switch

En masse curve/buckling of the original left side of the mid-region fold could explain the mid-region switch (see above). As described above, the switch refers to a changing of the position of the right and left sides of the mid-region fold, relative to the SMA. This could be explained by the following model.

The left side of the mid-region fold lengthens and buckles to the left and right of the middle colic vessel. As it does, the developing colon is forced superiorly and to the right (i.e. counter clockwise) under mechanical constraints imposed by the middle colic artery. In doing so, it overlaps mesentery at the same level in the right side of the fold, transiently generating a "U" shaped bend**.**

The anatomy of the bend is relevant to understanding the events that follow. The upper limb of the "U" is located in the original left side of the mid-region fold. It corresponds to a level in the left side. At that level, the hepatic region of the mesocolon continues as the splenic, across the middle colic artery. The lower limb of the "U" is located in mesentery of the original right side of the mid-region fold. Within the fold, it occurs at the same level as the lower limb, albeit in the right side of the fold. It corresponds to the junction between the mesoduodenum and mesojejunum.

The net torque force generated by continued lengthening of the colon, and buckling of adjoining mesocolon is such that the upper limb of the U pulls the lower limb around into alignment. As adjoining intestine at the duodenojejunal junction develops, this is displaced in tandem, from right to left of the midline. The developing colon continues to lengthen with the result that buckling progresses peripherally along the left side of the mid-region fold. Torque forces are transmitted to the right side of the mid-region fold. As a result, the periphery of the mid-region fold progressively unfurls from central to peripheral zones.

In summary, the shape of the intestine and mesentery (from duodenum to descending colon level) in the adult, is explained as follows. Early during development, the intestine lengthens within the mid-region and acquires an "Omega" shape (in the axial plane). At that stage, the mid-region mesentery forms a buckle (the mid-region fold). At that point in time a curve/buckle complex is apparent. With continued lengthening of the left side of the fold, the intestine coils, and changes from an omega to an alpha shaped conformation. In tandem with this the sides of the mesentery switch to form a spiral. The intestine and mesentery from duodenum to descending colon thus represents a single, large, coil/spiral complex.

17.4. Curve/buckle coupling and the apex of the mid-region fold

Early during development, the original apex of the mid-region fold is marked by the junction of the vitelline duct and developing endoderm. With obliteration of the duct, the apex is no longer mechanically constrained. It can then be displaced during the mid-region switch.

Normally, in the adult, the original apex is not apparent. In 2% of the general population it is retained in a vestigial form as a Meckel's diverticulum located 60 cm proximal to the ileocaecal junction. During left-sided curve/buckle coupling, the ileocaecal junction acquires an interim position immediately caudad the liver. With continued lengthening of colon and adjoining mesocolon, the junction is displaced further caudally into the right iliac fossa where it provides an apparent apex to the mid-region fold.

17.5. Curve/buckle coupling and the under surface of the mid-region

Counter-clockwise curve/buckling means mid-region opens upward and backward towards the liver In turn, torque forces pull the junction between mesoduodenum and mesojejunum downward and forward to the left of the midline. Continued curve/buckling along the left side of the mid-region leads to unfurling of the entire periphery of the mid-region. After unfurling of the periphery of the mid-region fold, the original undersurface of the mid-region is now upward facing. Adhesion of the mid-region fold (see above) means the upward facing surface ends by facing anteriorly. These events explain why the original undersurface of the mid-region fold is normally directly in view on entry into a human abdomen.

Supplementary Figure 1

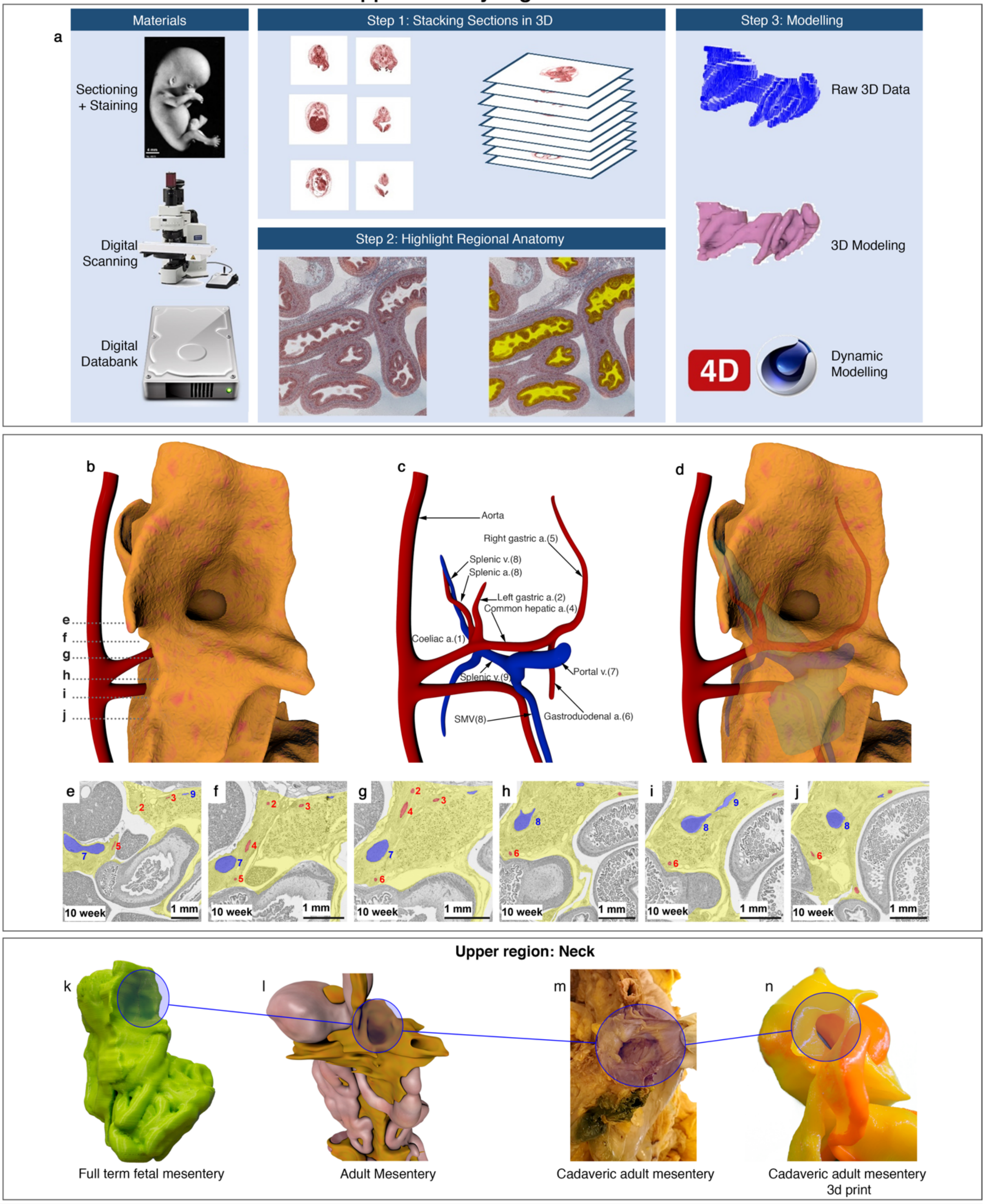

Supplementary Figure 1: Model generation and assessment: a, Overview of methodology used to reconstruct the mesentery and conjugate organs. **b**, Photographs demonstrating right lateral aspect of mesentery and vasculature **(b)**, vasculature, **(c)**, and vasculature and translucent mesentery **(d)**, at week ten of development. **e-j**, Photomicrographs demonstrating the histological appearance of mesentery and vasculature at levels indicated in **(b)**. Vessels are numbered as in **(b)**. Photographs of a 3D printed fetal mesentery **(k)**, *in vivo* adult *mesentery* **(l)**, and cadaveric mesentery **(m)**, and 3D printed adult *ex vivo* mesentery **(n)**.
The right lateral aspect of the upper region neck is apparent.

Supplementary Figure 2

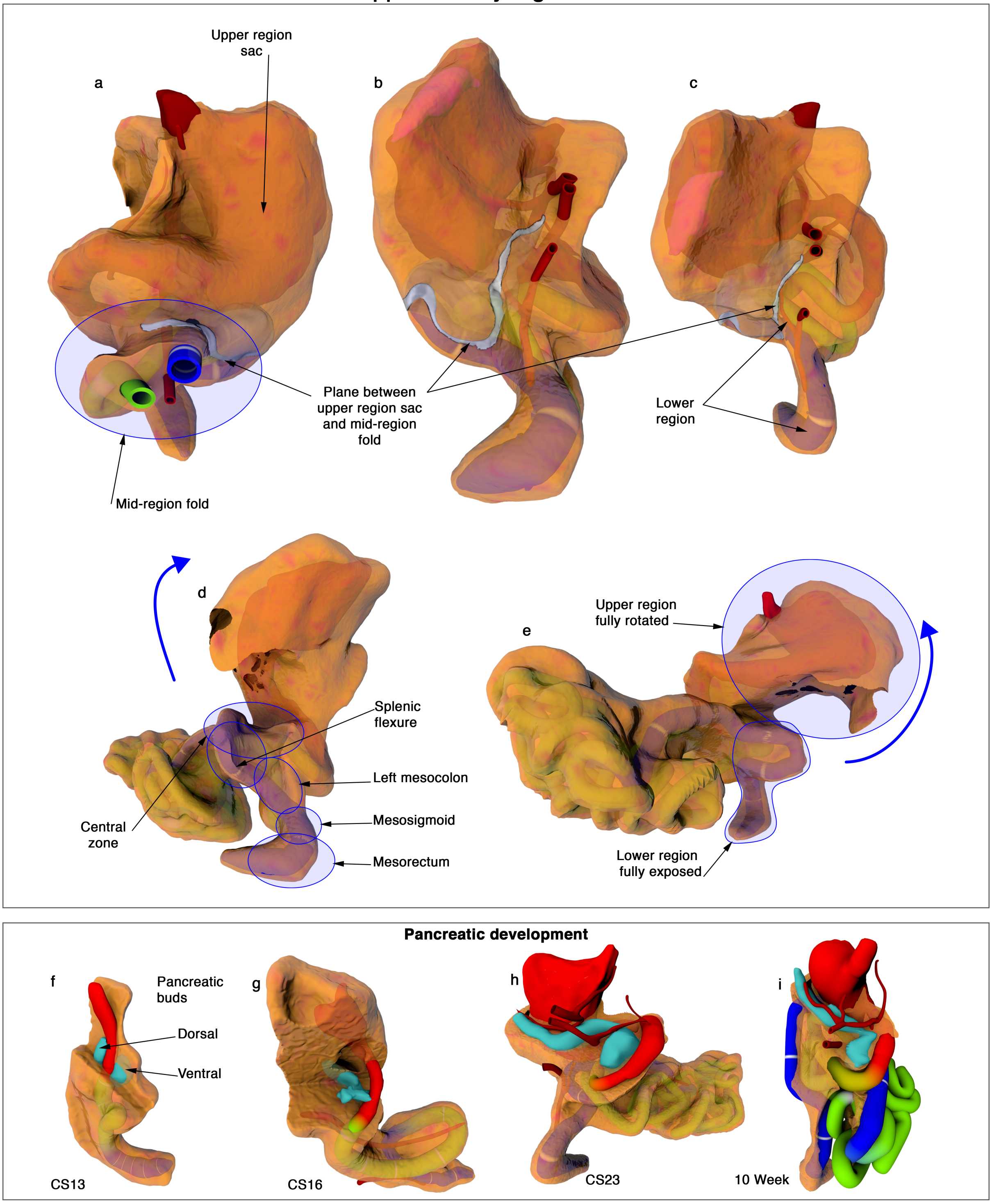

Supplementary Figure 2: Part a: mesentery and conjugate organs. a, Illustration of a reconstruction of the mesentery at Carnegie stage 19. The plane between the upper region body and mid region fold is colored grey. Illustrations demonstrating anterolateral **(b)** and posterior **(c)** aspects of the plane between upper and mid regions of the mesentery. Illustrations of **(d)** left posterolateral and **(e)** anterolateral aspects of the upper and mid-region after the upper region was digitally rotated off the mid-region. **f-I**, Panel of illustrations of the reconstructed mesentery (yellow) during development. Sections expose the developing intestine (red), pancreas (blue) and spleen (purple).

Supplementary Figure 3

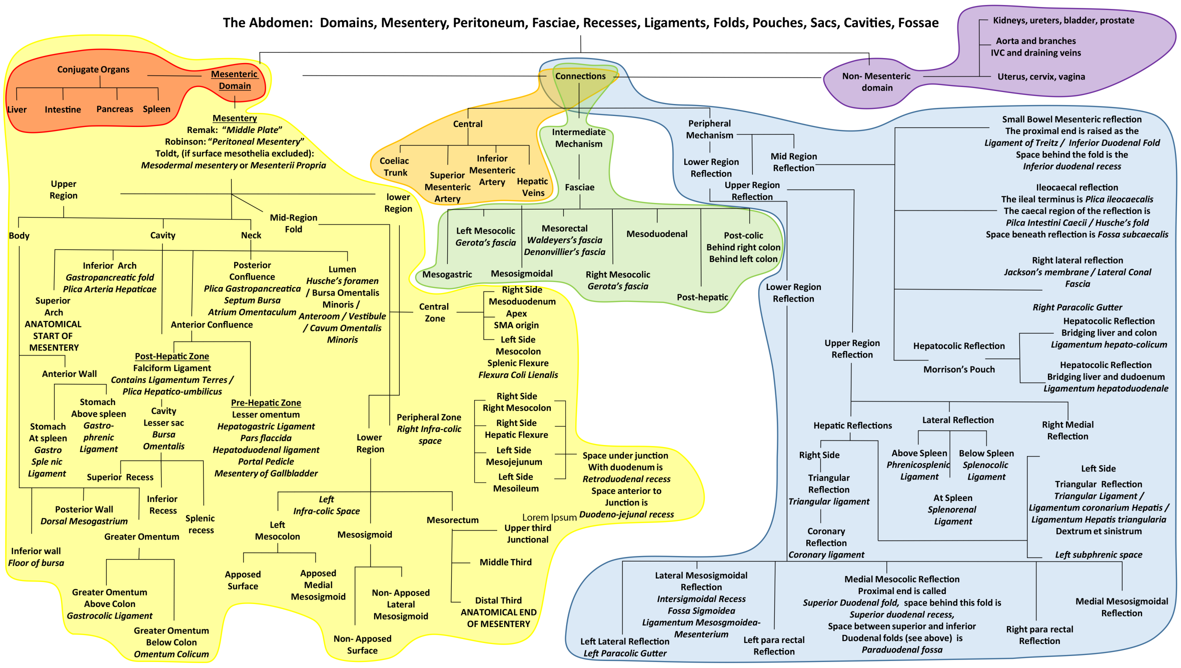

Supplementary Figure 3: Mesenteric model of abdominal anatomy: Schematic depiction of the position of the contents of the abdomen based on the mesenteric model of abdominal anatomy.
Color code –
yellow area represents the mesenteric domain; purple area represents the non-mesenteric domain; red area refers to conjugate organs of the mesentery; orange area refers to the central mechanisms of **connection** between domains; green area represents the **fascia** between domains; light blue area represents the peritoneal **connection** between domains.

Supplementary Figure 4

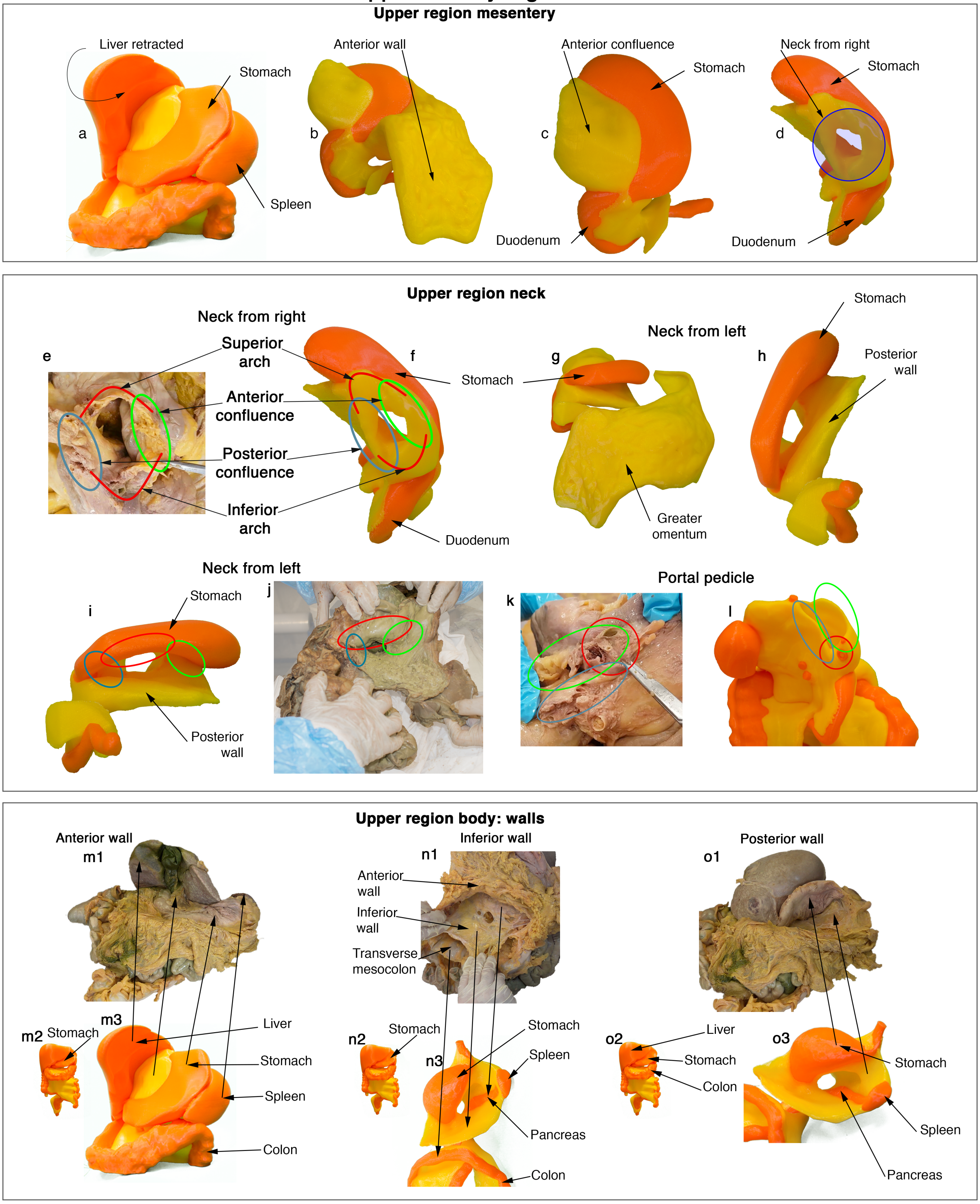

Supplementary Figure 4: Upper region: a-d, Panel of photographs of 3D printed upper region showing left lateral **(a)**, right anterolateral **(b)**, left anterolateral **(c)**, right lateral aspects **(d)**. Right lateral aspect of neck in *ex vivo* **(e)** and in 3D print **(f)** formats. Left lateral aspect of neck in 3D print **(g,h,i)** and *ex vivo* **(j)** formats. Right infero-lateral aspect of portal pedicle in *ex vivo* **(k)** and 3D **(l)** printed format. Panels demonstrating the anterior **(m1-3)**, posterior **(o1-3)** and inferior **(n1-3)** walls of the upper region in *ex vivo* and 3D printed formats.

Supplementary Figure 5

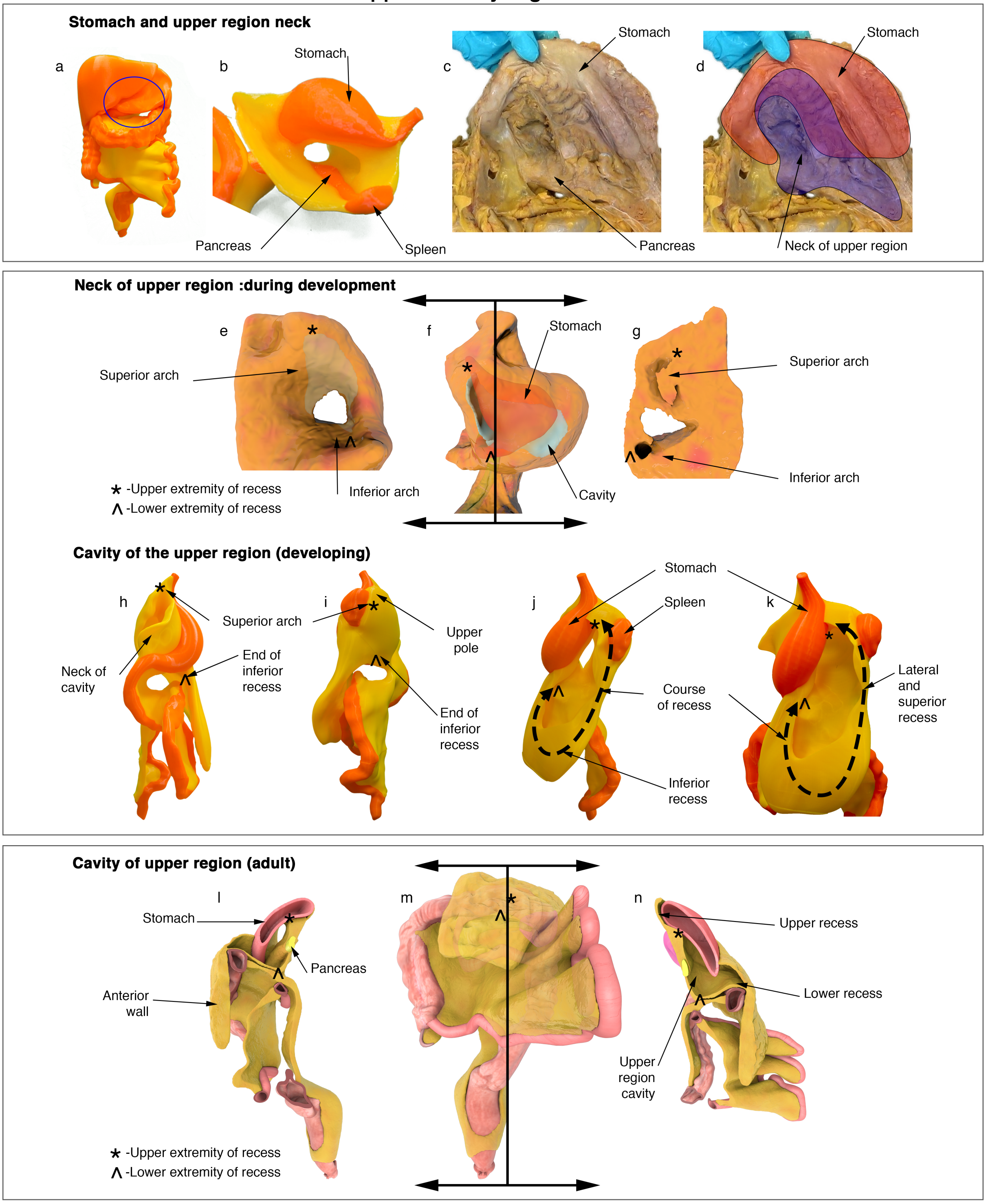

Supplementary Figure 5: Upper region cavity: a, Photograph of a 3D printed *ex vivo* mesentery. **b**, Photograph of a 3D printed upper region (anterior wall removed) showing the left lateral aspect of the stomach and neck. **c, d** Photographs of the left lateral aspect of the stomach and neck (neck (blue) and stomach (red)). **e-g**, Illustrations of the upper region neck in a digital reconstruction of the developing mesentery. **h-k**, Photographs of a 3D printed upper region during development, demonstrating the recess (dotted line). **l-n**, Illustrations of the upper region cavity in a digital reconstruction of the adult mesentery.

Supplementary Figure 6

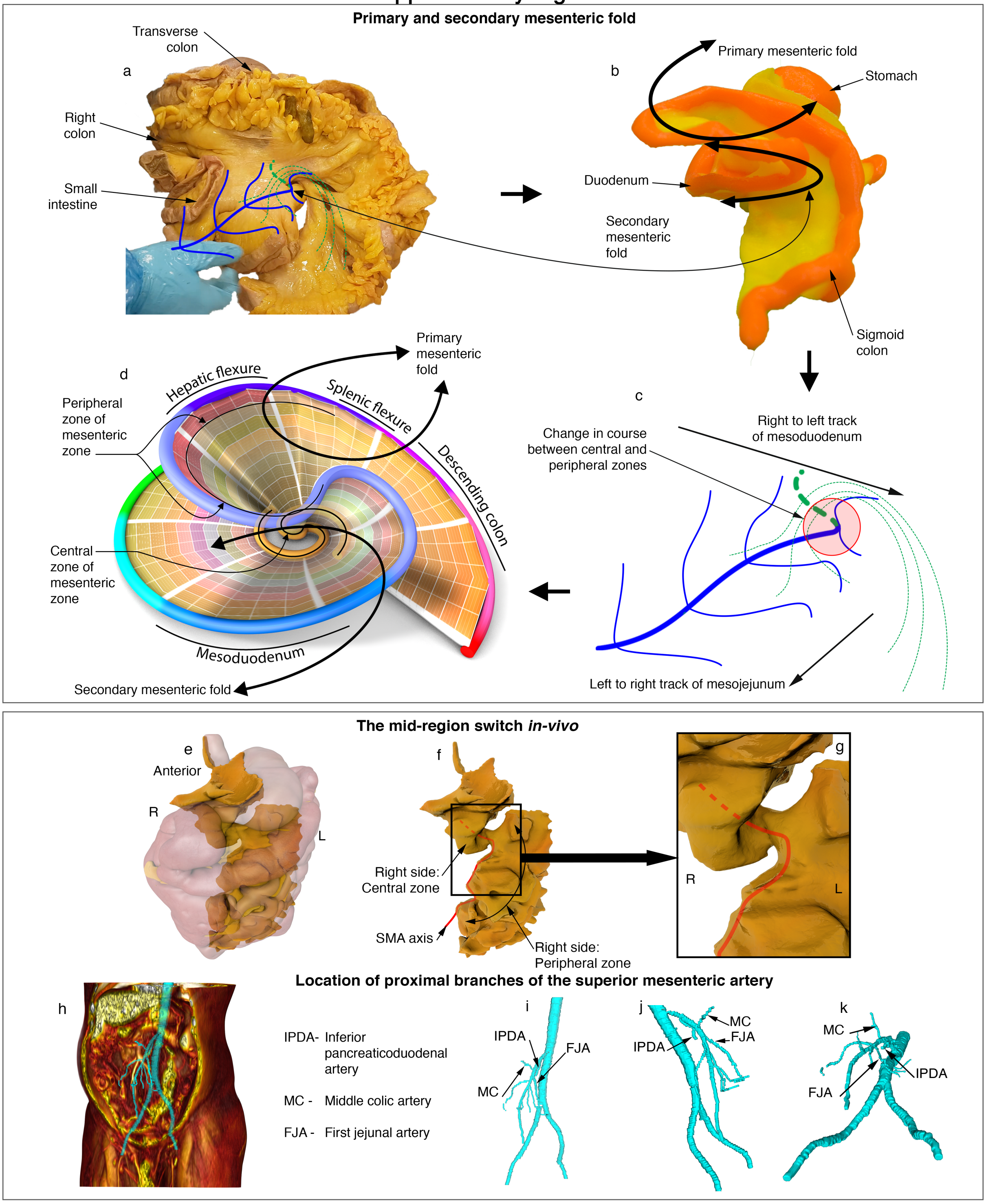

Supplementary Figure 6: Part a: Mesenteric folding: **a**, Photograph of the peripheral zone of the mid-region. Dotted green and continuous blue lines delineate the course of the right side of the mid-region, at central and peripheral zones respectively. **b**, Photograph of a 3D print demonstrating primary and secondary folding. **c**, Schematic demonstrating transition between central and peripheral zones. **d**, Schematic depiction of folding of the mesentery. **e-g**, Photographs of a digital reconstruction explaining the position of the right side of the mid-region relative to the superior mesenteric artery (SMA)(red). **h-k**, Panel demonstrating reconstructions of branches of the SMA.

Supplementary Figure 7

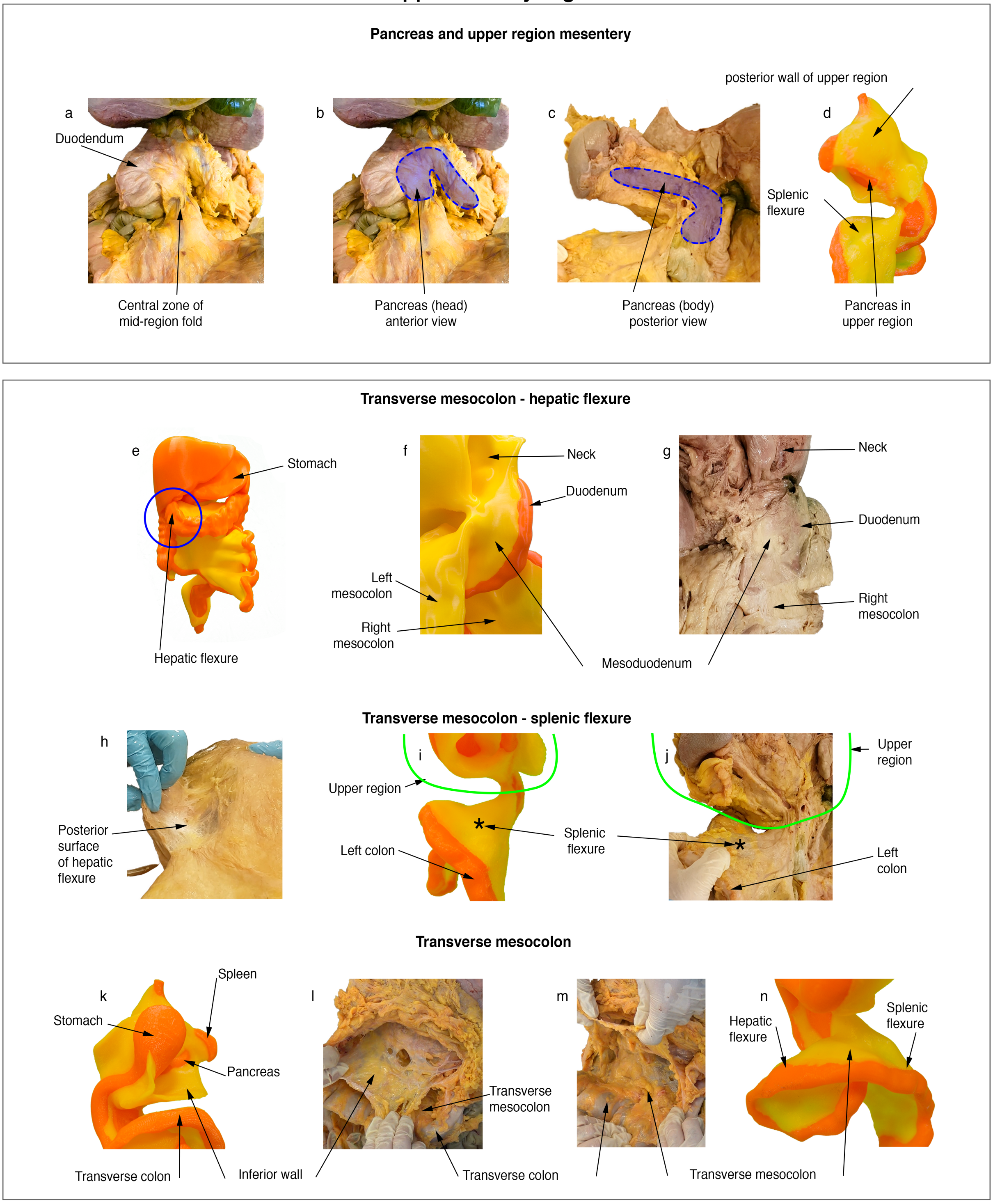

Supplementary Figure 7: Part a: The pancreas and flexures. The following photographs illustrate: antero-superior **(a,b)** and posterior **(c)** aspects of the central zone (pancreas/blue); **d**, posterior aspect of 3D printed upper region; **e**, anterior aspect of 3D printed mesentery; right posterolateral aspect of the mesoduodenum in a 3D printed **(f)** and *ex vivo* **(g)** mesentery; **h**, right anterolateral aspect of the mesoduodenum during separation of central and peripheral zones; left lateral aspect of the splenic flexure in 3D printed **(i)** and *ex vivo* **(j)** mesentery; **k-n**, inferior wall of upper region and transverse mesocolon.

Supplementary Figure 8

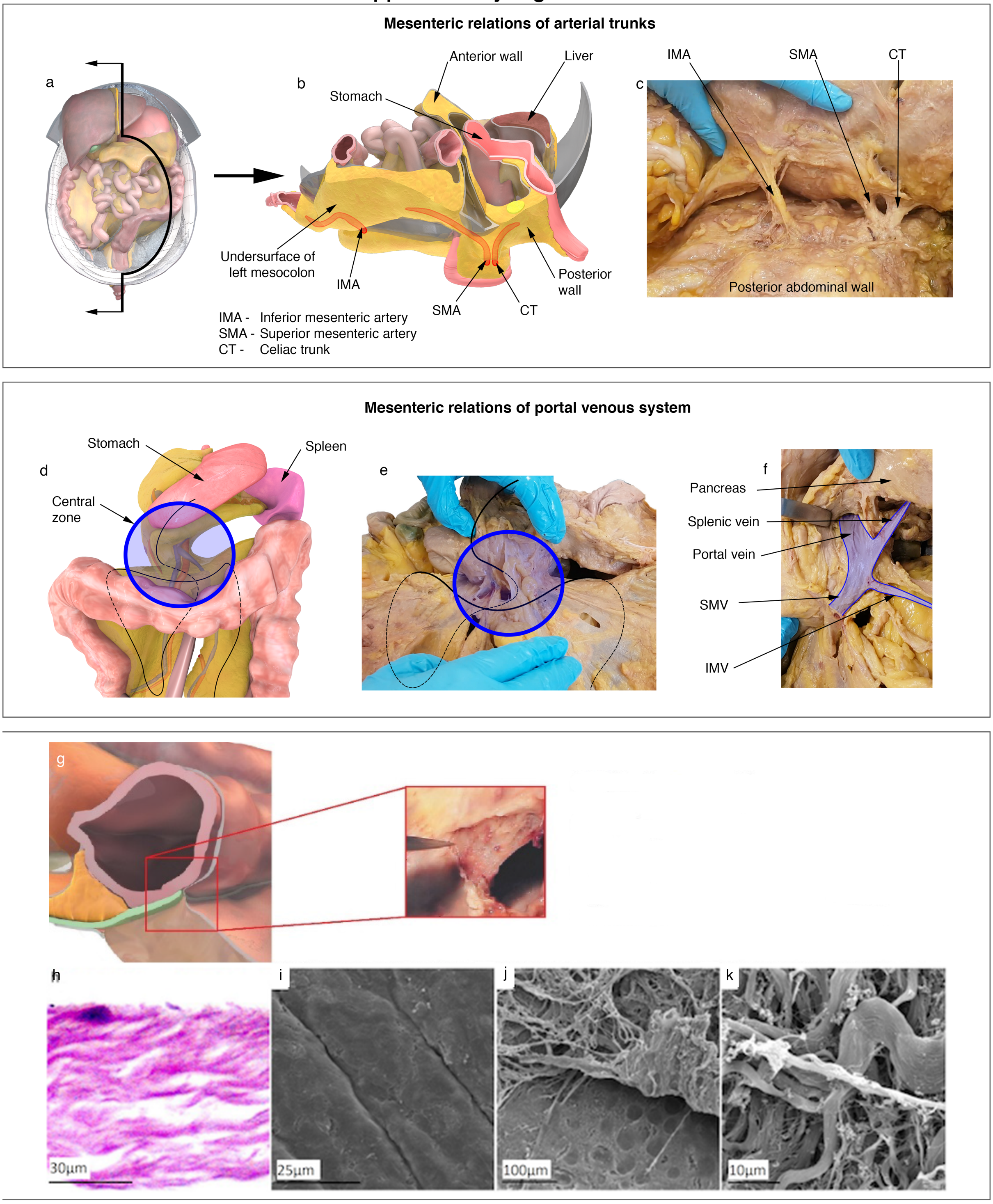

Supplementary Figure 8: Vascular connections of mesenteric domain: **a**, Illustration of the abdomen (as per the mesenteric model). Illustration of arterial connections in a digital reconstruction **(b)** and *in vivo* **(c).** **d**, Illustrations of the antero-superior aspect of the central zone in a digital reconstruction **(d)** and *in vivo* **(e)** and following exposure of portal venous system **(f)**. **g**, Photograph depicting sampling of the reflection. **h**, Photomicrograph of reflection stained with haematoxylin and eosin. **i**, Scanning electron microscopic photomicrographs of the reflection demonstrating the upper surface **(i)**, under surface **(j)** and under surface connective tissue **(k)**.

Supplementary Figure 9

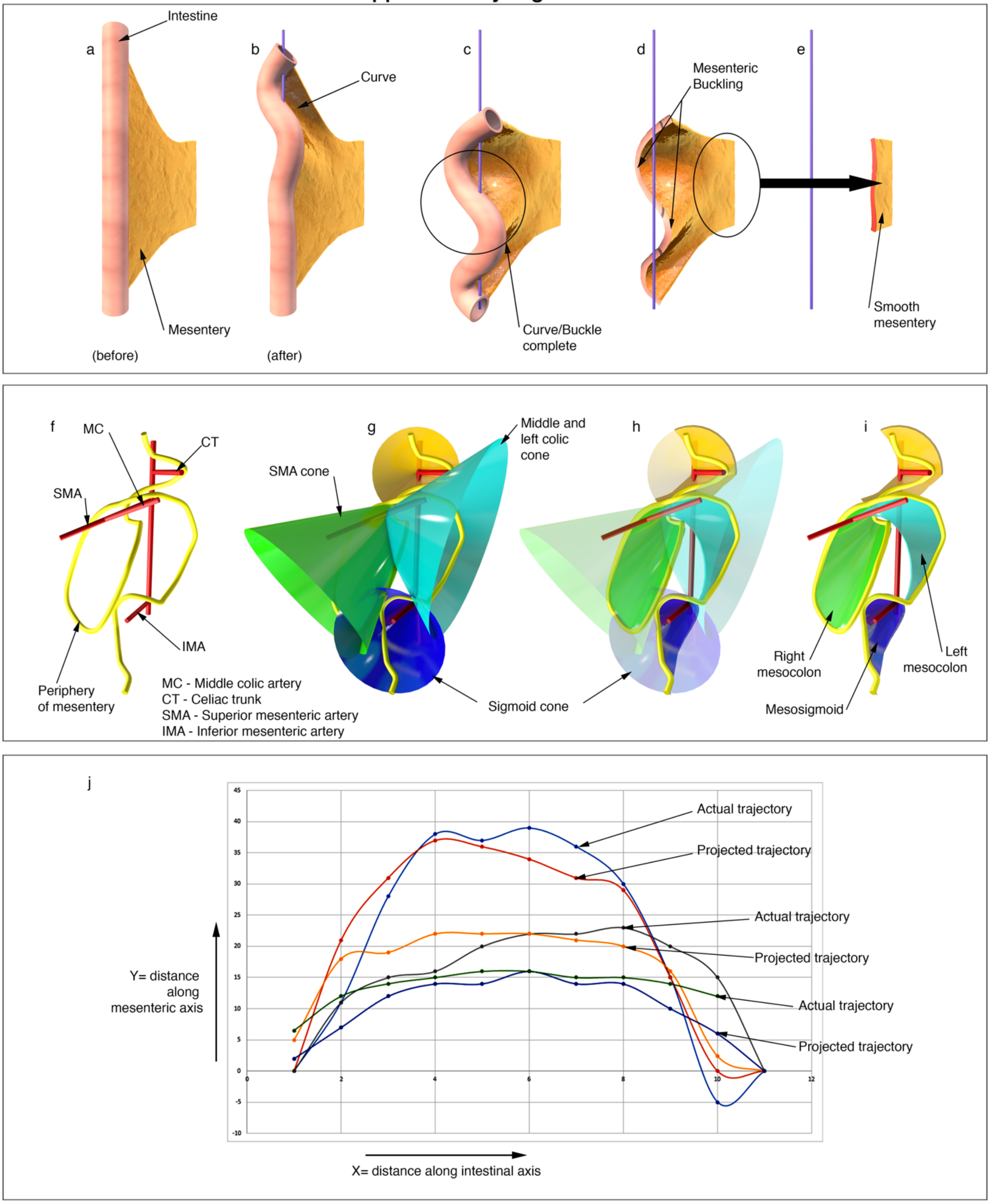

Supplementary Figure 9: Curve/buckle coupling: a-e, Panel demonstrating shapes acquired by intestine and adjoining mesentery as the intestine lengthens and the mesentery buckles (i.e. curve/buckle coupling). **f**, Schematic depiction of the periphery of the mesentery (yellow tube) and major vessels (red bars). **g**, Schematic depiction of conal model. **h,i** Schematic depictions of the mesentery in the conal model. **j**, Line graph demonstrating the trajectory of the periphery of the mesentery. Each pair of lines represents the actual trajectory of the periphery and the trajectory as predicted by the conal model.

References

1. De Bakker BS, De Jong KH, Hagoort J, et al. An interactive three-dimensional digital atlas and quantitative database of human development. *Science (80-)*. 2016;354(6315):aag0053. doi:10.1126/science.aag0053
2. Hansma P. The evolution of the autopsy. *Acad Forensic Pathol*. 2015;5(4):638-649.
3. Nagai H. Configurational anatomy of the pancreas: Its surgical relevance from ontogenetic and comparative-anatomical viewpoints. *J Hepatobiliary Pancreat Surg*. 2003;10(1):48-56. doi:10.1007/s10534-002-0796-6
4. Nakamura T, Yamada S, Funatomi T, Takakuwa T, Shinohara H, Sakai Y. Three-dimensional morphogenesis of the omental bursa from four recesses in staged human embryos. *J Anat*. 2020.
5. Bardeen CR. The critical period in the development of the intestines. *Am J Anat*. 1914;16(4):427-445.
6. García-Arrarás JE, Bello SA, Malavez S. The mesentery as the epicenter for intestinal regeneration. *Semin Cell Dev Biol*. 2019;92:45-54. doi:10.1016/j.semcdb.2018.09.001
7. Murray G, García-Arrarás JE. Myogenesis during holothurian intestinal regeneration. *Cell Tissue Res*. 2004;318(3):515-524. doi:10.1007/s00441-004-0978-3
8. Coffey JC, O'Leary DP. The mesentery: structure, function, and role in disease. *Lancet Gastroenterol Hepatol*. 2016;1(3):238-247. doi:10.1016/S2468-1253(16)30026-7
9. Byrnes KG, Walsh D, Lewton-Brain P, McDermott K, Coffey JC. Anatomy of the mesentery: Historical development and recent advances. *Semin Cell Dev Biol*. 2019;92:4-11. doi:10.1016/j.semcdb.2018.10.003
10. Toldt C. Bau und Wachsthumsveränderungen der Gekröse des menschlichen Darmkanales. *Denkschrdmathnaturwissensch*. 1879;41:56.
11. Robinson B. The Morphology of the Mesenterial Development of the Vertebrate Digestive Tract. *J Anat Physiol*. 1899;33(Pt 3):434-43470.
12. Shinohara H, Kurahashi Y, Haruta S, Ishida Y, Sasako M. Universalization of the operative strategy by systematic mesogastric excision for stomach cancer with that for total mesorectal excision and complete mesocolic excision colorectal counterparts. *Ann Gastroenterol Surg*. 2018;2(1):28-36. doi:10.1002/ags3.12048
13. Moore KL, Agur AMR, Dalley AF, Moore KL. *Essential Clinical Anatomy*. Wolters Kluwer Health,; 2015.
14. Standring S. *Gray's Anatomy: The Anatomical Basis of Clinical Practice*. Elsevier Limited; 2016. https://books.google.ie/books?id=LjP9rQEACAAJ.
15. Hansen JT. *Netter's Clinical Anatomy E-Book*. Elsevier Health Sciences; 2017.
16. Culligan K, Walsh S, Dunne C, et al. The mesocolon: A histological and electron microscopic characterization of the mesenteric attachment of the colon prior to and after surgical mobilization. *Ann Surg*. 2014;260(6):1048-1056. doi:10.1097/SLA.0000000000000323

17. Savin T, Kurpios NA, Shyer AE, et al. On the growth and form of the gut. *Nature*. 2011;476(7358):57-63. doi:10.1038/nature10277

18. Nerurkar NL, Mahadevan L, Tabin CJ. BMP signaling controls buckling forces to modulate looping morphogenesis of the gut. *Proc Natl Acad Sci U S A*. 2017;114(9):2277-2282. doi:10.1073/pnas.1700307114

19. Chevalier NR, De Witte TM, Cornelissen AJM, Dufour S, Proux-Gillardeaux V, Asnacios A. Mechanical Tension Drives Elongational Growth of the Embryonic Gut. *Sci Rep*. 2018;8(1):5995. doi:10.1038/s41598-018-24368-1

20. Davis A, Amin NM, Johnson C, Bagley K, Ghashghaei HT, Nascone-Yoder N. Stomach curvature is generated by left-right asymmetric gut morphogenesis. *Dev*. 2017;144(8):1477-1483. doi:10.1242/dev.143701
