# Supplementary material for: The development and structure of the mesentery
Source: Commun Biol. 2021 Aug 18;4:982. doi: 10.1038/s42003-021-02496-1 (PMC8373875; doi:10.1038/s42003-021-02496-1)
Supplement: Supplementary file 2 — Reporting Summary [file 42003_2021_2496_MOESM2_ESM.pdf]

nature research

Corresponding author(s): J Calvin Coffey

Last updated by author(s): Jul 4, 2021

Reporting Summary

Nature Research wishes to improve the reproducibility of the work that we publish. This form provides structure for consistency and transparency in reporting. For further information on Nature Research policies, see our Editorial Policies and the Editorial Policy Checklist.

Statistics

For all statistical analyses, confirm that the following items are present in the figure legend, table legend, main text, or Methods section.

n/a	Confirmed	
☒	☐	The exact sample size (n) for each experimental group/condition, given as a discrete number and unit of measurement
☒	☐	A statement on whether measurements were taken from distinct samples or whether the same sample was measured repeatedly
☒	☐	The statistical test(s) used AND whether they are one- or two-sided<br>*Only common tests should be described solely by name; describe more complex techniques in the Methods section.*
☒	☐	A description of all covariates tested
☒	☐	A description of any assumptions or corrections, such as tests of normality and adjustment for multiple comparisons
☒	☐	A full description of the statistical parameters including central tendency (e.g. means) or other basic estimates (e.g. regression coefficient) AND variation (e.g. standard deviation) or associated estimates of uncertainty (e.g. confidence intervals)
☒	☐	For null hypothesis testing, the test statistic (e.g. F, t, r) with confidence intervals, effect sizes, degrees of freedom and P value noted<br>*Give P values as exact values whenever suitable.*
☒	☐	For Bayesian analysis, information on the choice of priors and Markov chain Monte Carlo settings
☒	☐	For hierarchical and complex designs, identification of the appropriate level for tests and full reporting of outcomes
☒	☐	Estimates of effect sizes (e.g. Cohen's d, Pearson's r), indicating how they were calculated

Our web collection on statistics for biologists contains articles on many of the points above.

Software and code

Policy information about availability of computer code

Data collection	Data collection was performed on ImageJ2 (v1.51). Analysis was performed on a windows 10 software system. Reconstruction was performed within the TRAKEM2 plugin within ImageJ2.
Data analysis	Cinema4D (version 10.0, Maxon Computer GmbH, Germany) was used for modeling and quantitative extraction of data from reconstructions. ZBrush (version 3.5 R3; Pixologic, California, USA) was used to generate 3D models. Image brightness and histogram adjustment was performed in Adobe Lightroom (version 8.1, Adobe®, San Jose, California, United States).

For manuscripts utilizing custom algorithms or software that are central to the research but not yet described in published literature, software must be made available to editors and reviewers. We strongly encourage code deposition in a community repository (e.g. GitHub). See the Nature Research guidelines for submitting code & software for further information.

Data

Policy information about availability of data

All manuscripts must include a data availability statement. This statement should provide the following information, where applicable:

- Accession codes, unique identifiers, or web links for publicly available datasets
- A list of figures that have associated raw data
- A description of any restrictions on data availability

Data from reconstructions and dissections that support the findings of the study are available in the Atlas of the mesentery (http://mesentery.ie). The data that support the findings of the study are available from the corresponding author upon request.

Field-specific reporting

Please select the one below that is the best fit for your research. If you are not sure, read the appropriate sections before making your selection.

- [x] Life sciences
- [ ] Behavioural & social sciences
- [ ] Ecological, evolutionary & environmental sciences

For a reference copy of the document with all sections, see nature.com/documents/nr-reporting-summary-flat.pdf

Life sciences study design

All studies must disclose on these points even when the disclosure is negative.

Sample size	Dissection performed on six adult cadavers and individual dissections performed on X number of species. CT reconstructions performed on 10 adult patients. Developmental reconstructions performed on digitized serial sections from 12 human embryos/foetuses. Scanning electron microscopy was performed on tissue harvested from three patients.
Data exclusions	Gastrointestinal diseases frequently disrupt the position and shape of abdominal viscera and confound interpretations of anatomical shape. Cadavers were therefore evaluated, in conjunction with available medical records, for the presence of structural anomalies within the abdomen. Cadavers with any previously documented abdominal disease or apparent structural abnormalities within the abdominal cavity were excluded. All cadavers included in this study were male (due to availability rather than exclusion).
Replication	Dedicated sections to replication are included in methodology "Gastrointestinal diseases frequently disrupt the position and shape of abdominal viscera and confound interpretations of anatomical shape." and "Reconstruction of in vivo mesenteric vasculature using abdominal cross sectional imaging".
Randomization	Not applicable.
Blinding	Not applicable (single-armed, observational study).

Reporting for specific materials, systems and methods

We require information from authors about some types of materials, experimental systems and methods used in many studies. Here, indicate whether each material, system or method listed is relevant to your study. If you are not sure if a list item applies to your research, read the appropriate section before selecting a response.

Materials & experimental systems

n/a	Involved in the study	
x		Antibodies
x		Eukaryotic cell lines
x		Palaeontology and archaeology
	x	Animals and other organisms
	x	Human research participants
x		Clinical data
x		Dual use research of concern

Methods

n/a	Involved in the study	
x		ChIP-seq
x		Flow cytometry
x		MRI-based neuroimaging

Animals and other organisms

Policy information about studies involving animals; ARRIVE guidelines recommended for reporting animal research

Laboratory animals	No laboratory experimental animals were included in the study. Fresh tissue dissections were performed on a wide range of species as outlined in supplement on (http://mesentery.ie - section: comparative anatomy).
Wild animals	Details of individual species used in the comparative anatomy study are outlined in full detail on the study repository (http://mesentery.ie). All animals were euthanized in accordance with conditions outlined in local ethical approval application on grounds deemed humane. All experimental procedures described in this work were approved under University College Dublin animal research ethics committee. The principal method of euthanasia was by lethal injection with pentobarbitone sodium. Experiments were conducted under experimental license from the Department of Health in accordance with the cruelty to animal act 1876 and the European Communities (Amendments of Cruelty to Animal Act, 1876) Regulations (1994).
Field-collected samples	The study did not involve samples field-collected samples.
Ethics oversight	Approval for this study was obtained from the local research ethics committee (University of Limerick, Limerick, Ireland). Ethical approval for data received from international collaborators was obtained from their respective local research ethics committees.

Note that full information on the approval of the study protocol must also be provided in the manuscript.

Human research participants

Policy information about studies involving human research participants

Population characteristics	All human cadavers were male. Cadavers were therefore evaluated, in conjunction with available medical records, for the presence of structural anomalies within the abdomen. Cadavers with any previously documented abdominal disease or apparent structural abnormalities within the abdominal cavity were excluded.
Recruitment	Dissections were performed on human cadavers when availability permitted for research purposes. Recruitment occurred over a two year period (January 2018 to December 2019).
Ethics oversight	Approval for this study was obtained from the local research ethics committee (University of Limerick, Limerick, Ireland). Ethical approval for data received from international collaborators was obtained from their respective local research ethics committees. National legislation (Anatomy Act, Ireland 1832; Anatomy Act, Ireland 1871; Health Order, Ireland 1949; and Medical practitioners act, Ireland, 2007) governed the use of cadavers bequeathed to the National University of Ireland, Galway.

Note that full information on the approval of the study protocol must also be provided in the manuscript.
