# Supplementary material for: The development and structure of the mesentery
Source: Commun Biol. 2021 Aug 18;4:982. doi: 10.1038/s42003-021-02496-1 (PMC8373875; doi:10.1038/s42003-021-02496-1)
Supplement: Supplementary file 5 — Description of Additional Supplementary Files [file 42003_2021_2496_MOESM5_ESM.pdf]

Description of Additional Supplementary Files

File name: Supplementary Data 1
Description: Data summarising distances for points along the intestine (i.e. the periphery of the mesentery). x_a and y_a values correspond to actual distances along the mesenteric vasculature and intestine respectively. The y_p value corresponds to a predicted "y" value, if the periphery of the mesentery were to follow an elliptical course. The 1, 2 and 3 designations refer to measurements on an *ex vivo* mesentery, *in vivo* mesentery and a 3D model of the mesentery, respectively.

File name: Supplementary Data 2
Description: Data set for histograms in figure 2.
